# Cardiomyocyte-derived small extracellular vesicles can signal eNOS activation in cardiac microvascular endothelial cells to protect against Ischemia/Reperfusion injury

**DOI:** 10.7150/thno.43163

**Published:** 2020-09-23

**Authors:** Guihao Chen, Chuansheng Xu, Thomas G. Gillette, Tongyi Huang, Peisen Huang, Qing Li, Xiangdong Li, Qinfeng Li, Yu Ning, Ruijie Tang, Cunrong Huang, Yuyan Xiong, Xiaqiu Tian, Jun Xu, Junyan Xu, Liping Chang, Cong Wei, Chen Jin, Joseph A. Hill, Yuejin Yang

**Affiliations:** 1 State Key Laboratory of Cardiovascular Disease, Department of Cardiology, Fuwai Hospital, National Center for Cardiovascular Diseases, Chinese Academy of Medical Sciences and Peking Union Medical College, Beijing 100037, China; 2 Department of Internal Medicine (Cardiology), University of Texas Southwestern Medical Center, Dallas, TX 75390-8573, USA; 3 Department of Medical Ultrasound, Institute of Diagnostic and Interventional Ultrasound, First Affiliated Hospital, Sun Yat-Sen University, Guangzhou, Guangdong 510080, China; 4 Department of Cardiology, Biomedical Research (Therapy) Center, Sir Run Run Shaw Hospital, School of Medicine, Zhejiang University, Hangzhou 310020, China; 5 Center for Cardiac Intensive Care, Beijing Anzhen Hospital, Capital Medical University, Beijing Institute of Heart, Lung and Blood Vessel Diseases, Beijing 100029, China; 6 Yiling Hospital of Hebei Medical University, National Key Laboratory of Collateral Disease Research and Innovative Chinese Medicine, Shijiazhuang, Hebei 050035, China; 7 National Key Laboratory of Collateral Disease Research and Innovative Chinese Medicine, Key Laboratory of State Administration of TCM (Cardio-Cerebral Vessel Collateral Disease), Shijiazhuang, Hebei 050035, China

**Keywords:** Cardioprotection, tongxinluo, cardiomyocytes, endothelial cells, crosstalk

## Abstract

**Rationale:** The crosstalk between cardiac microvascular endothelial cells (CMECs) and cardiomyocytes (CMs) has emerged as a key component in the development of, and protection against, cardiac diseases. For example, activation of endothelial nitric oxide synthase (eNOS) in CMECs, by therapeutic strategies such as ischemic preconditioning, plays a critical role in the protection against myocardial ischemia/reperfusion (I/R) injury. However, much less is known about the signals produced by CMs that are able to regulate CMEC biology. Here we uncovered one such mechanism using Tongxinluo (TXL), a traditional Chinese medicine, that alleviates myocardial ischemia/reperfusion (I/R) injury by activating CMEC eNOS. The aim of our study is to identify the signals produced by CMs that can regulate CMEC biology during I/R.

**Methods:** *Ex vivo, in vivo,* and* in vitro* settings of ischemia-reperfusion were used in our study, with the protective signaling pathways activated in CMECs identified using genetic inhibition (p70s6k1 siRNA, miR-145-5p mimics, etc.), chemical inhibitors (the eNOS inhibitor, L-NNA, and the small extracellular vesicles (sEVs) inhibitor, GW4869) and Western blot analyses. TritonX-100 at a dose of 0.125% was utilized to inactivate the eNOS activity in endothelium to investigate the role of CMEC-derived eNOS in TXL-induced cardioprotection.

**Results:** We found that while CMEC-derived eNOS activity was required for the cardioprotection of TXL, activation of eNOS in CMECs by TXL did not occur directly. Instead, eNOS activation in CMECs required a crosstalk between CMs and CMECs through the uptake of CM-derived sEVs. We further demonstrate that TXL induced CM-sEVs contain increased levels of Long Intergenic Non-Protein Coding RNA, Regulator Of Reprogramming (Linc-ROR). Upon uptake into CMECs, linc-ROR downregulates its target miR-145-5p leading to activation of the eNOS pathway by facilitating the expression of p70s6k1 in these cells. The activation of CMEC-derived eNOS works to increase survival in both the CMECs and the CMs themselves.

**Conclusions:** These data uncover a mechanism by which the crosstalk between CMs and CMECs leads to the increased survival of the heart after I/R injury and point to a new therapeutic target for the blunting of myocardial I/R injury.

## Introduction

Coronary heart disease is the leading cause of death worldwide 1. Timely and successful reperfusion, such as primary percutaneous coronary intervention, is the most effective treatment for patients suffering from acute myocardial infarction (AMI) 2. However, aside from the ischemic injury, restoration of blood flow to endangered cardiomyocytes *per se* can paradoxically result in injuries to the myocardium, a phenomenon called reperfusion injury. Therefore, it is theoretically feasible that the prognosis of patients with AMI will be further improved if reperfusion injury can be alleviated or even prevented 3. Although numerous strategies such as ischemic preconditioning have been reported to exert robust protective effects in animal model of I/R injury in the past 30 years, the translation of these beneficial therapies into clinical practice has been disappointing 4. Consequently, new therapeutics should still be tested to determine whether they can reduce I/R injury or can be used to elucidate the mechanisms of I/R injury.

In hearts, cardiac microvascular endothelial cells (CMECs), which are located in the capillaries, closely surround CMs, with a short distance between these two types of cells (approximately 1-2 μm) 5. Anatomically, several CMECs border one cardiomyocyte and can be regarded as a working “perfusion unit”. In these “perfusion units”, the close proximity between CMs and CMECs guarantees the important role of the crosstalk between these two types of cells, in both physiological (normal cardiac development) and pathophysiological conditions (ischemia, remodelling and metabolic dysfunction) in the heart 6. Accumulating evidence suggests that the crosstalk between CMECs and CMs may be instrumental in the progression of myocardial I/R injury and thus, is a new potential therapeutic target for mitigating myocardial I/R injury. For instance, endothelium-derived neuregulin has been demonstrated to protect CMs from I/R-induced injury 7, and sEVs secreted from endothelial cells after ischemic preconditioning can make CMs less vulnerable to injury induced by H/R (I/R) 8. Furthermore, cardioprotection conferred by exercise training is dependent on the activation of eNOS in endothelium 9. Although the role of endothelial cells in protecting CMs during I/R is clear, few studies have investigated whether signals produced by CMs are able to impact CMECs and thereby reduce myocardial I/R injury.

Early preconditioning, which is implemented before a prolonged period of ischemia, is a physiological adaptive phenomenon where brief episodes of ischemia interspersed with reperfusion (ischemic preconditioning), or organs are exposed to certain pharmacological agents (pharmacological preconditioning), such as isoflurane. The cardioprotective effect of early preconditioning has been confirmed in dozens of studies, and is proved to be largely mediated by the activation of eNOS pathway 10, 11. eNOS is an enzyme primarily responsible for the generation of NO in the vascular endothelium 12. In hearts, endothelial cells 13 and other non-endothelial cells such as cardiomyocytes 14 and fibroblasts 15 express eNOS. Our early studies have demonstrated that preconditioning the hearts with Tongxinluo (TXL), a traditional Chinese medicine, can protect the hearts from I/R injury by activating the eNOS pathway 16, 17. However, it remains unknown that whether the infarct-sparing effect of TXL is mediated by eNOS in endothelial cells, and how TXL activates endothelial eNOS. When examining this question, we uncovered a mechanism whereby crosstalk between CMs and CMECs impact CMECs to reduce myocardial I/R injury.

We show that preconditioning the hearts with TXL leads to the release of small extracellular vesicles (sEVs) from CMs that then stimulate eNOS activation in CMECs. These studies suggest that targeting this cellular crosstalk may provide a novel approach for the reduction of myocardial I/R injury.

## Materials and Methods

### Preparation of TXL Solution for the *In Vitro* Experiments

A solution of TXL ultrafine powder (Lot Number: 071201; Shijiazhuang Yiling Pharmaceutical Co., Shijiazhuang, China) was made as described previously 18. In brief, after TXL was dissolved in serum-free Dulbecco's modified Eagle's medium (DMEM; Life Technologies, Grand Island, NY, USA), the suspension was sonicated for 30 min and then centrifuged at 2,500 rpm for 15 min. A 0.22-µm filter was used to filter the supernatant to prepare a sterile TXL solution.

### Animals

Male Sprague Dawley rats (220-250 g) were utilized in our experiments. Animal studies were performed in accordance with the “Guide for the Care and Use of Laboratory Animals” issued by the US National Institutes of Health (Bethesda, MD, USA, NIH Publication No. 85-23, revised 1996) and the “Regulation to the Care and Use of Experimental Animals” of the Beijing Council on Animal Care (1996). The experimental protocol was reviewed and approved by the Care of Experimental Animals Committee of Fuwai Hospital.

### Establishment of an *in vivo* Myocardial I/R Injury Model and infarct size measurement

Male Sprague Dawley rats were anesthetized with sodium pentobarbital (50 mg/kg, intraperitoneally) before endotracheal intubation. I/R was induced by ligating the left anterior descending artery (LAD) for 45 min, followed by loosening the ligature for 3 hours, as described previously 19. Rats were randomized to: (1) Sham group, in which LAD was encircled by a suture but not occluded, and rats were administered saline by gavage 1 h prior to I/R; (2) I/R group, in which rats were administered saline by gavage 1 h prior to I/R; (3) I/R + TXL group, in which rats were administered TXL dissolved in saline (0.4 g/kg, an equivalent dose to that used clinically in humans) by gavage 1 h prior to I/R; and (4) I/R + TXL + GW4869 (Selleck, Houston, TX, USA) group, in which the rats were administered GW4869 (1 mg/kg) intraperitoneally every 24 h for 3 days prior to I/R, as described previously 20, 21, in addition to receiving the same TXL treatment as the TXL group. GW4869 was dissolved in saline with 2.5% DMSO. The same volume of 2.5% DMSO saline buffer was injected as a vehicle control in groups where GW4869 was not given.

Myocardial infarct sizes were assessed as previously described 18. Briefly, after reperfusion, the suture was re-tied to ligate the LAD again, and 1 mL 2% Evans Blue dye (Sigma, Darmstadt, Germany) was injected into the thoracic aorta to delineate the ischemic area (area-at-risk, AAR) and the non-ischemic area (Evans blue perfused region). Then the heart was harvested and stored at -80°C overnight. On the second day, frozen ventricles were cut into 5 or 6 slices and incubated in 1% 2, 3, 5-triphenyltetrazolium chloride (TTC, Amresco, USA) for 15 min at 37°C. After staining, slices were incubated in 10% formalin to increase the contrast between dyed and undyed tissues. Ischemic but viable tissue will be stained red and the infarcted area (IA) will be stained pale white. ImageJ software (National Institutes of Health) was used to measure the IA, AAR, and total cross-sectional heart area (TA). A percentage of AAR (IA/AAR) or TA (AAR/TA) was used to express the infarct size and ischemic area, respectively.

### Detection of microcirculatory perfusion

To determine microcirculatory blood flow during myocardial (M) I/R, rats were anesthetized using pentobarbital sodium, intubated and ventilated with a positive-pressure respirator. After removing the thoracic muscle, the thoracic cage was opened via thoracotomy through the 4th intercostal space on both sides and the mid-sternum. The heart was then completely exposed by cutting 2-4 ribs on both sides and fixing the free anterior thorax wall towards the rat's head with a forcep. To induce myocardial ischemia, the LAD coronary artery was ligated with a 6-0 silk suture 2-3 mm from the origin. 45 minutes later, the ligature was removed to re-perfuse the ischemic myocardium. To monitor microcirculatory perfusion, hearts were scanned using laser Doppler flowmetry (PeriCam PSI System, Perimed, Sweden) at baseline (prior to ligation), and 0, 10, 20, 30 and 40 mins post-reperfusion. Left ventricular blood flow at the ischemic part was determined and analyzed blindly.

### Langendorff model, study design and infarct size measurement

After being heparinized (3 IU/g BW) and anesthetized with sodium pentobarbital (50 mg/kg, intraperitoneally), rat hearts were rapidly removed (the procedure time from opening the chest to heart excision was less than 2 min). Then the aorta was immediately connected to a standard Langendorff apparatus and perfused with Krebs-Henseleit (K-H) buffer solution (pH 7.4: in mM: NaCl 118; KCl 4.7; CaCl_2_ 2.0; MgSO_4_ 1.2; NaHCO_3_ 25; KH_2_PO_4_ 1.2; glucose 11.1) gassed with 95% O_2_ and 5% CO_2_ during the perfusion, at a constant flow of 10 mL/min at 37°C. A pulmonary arteriotomy was performed to drain coronary effluent. To establish a global I/R model, the perfusion pump was switched off for 40 min to stop coronary flow and switched on for 60 min to re-establish perfusion.

Rats were randomized to: (1) I/R; (2) I/R +TXL; (3) I/R+TXL+TritonX-100; and (4) I/R+TXL+L-NNA. TXL (400 μg/mL) was added into the perfusion fluid before ischemia and throughout reperfusion in the presence or absence of L-NNA (0.1 mM) or TritonX-100 (0.125%). L-NNA is a competitive inhibitor with selectivity for NOS. TritonX-100 at a low dose, as demonstrated before 22, can be used to inactivate the eNOS activity and decrease NO synthesis in endothelium, without losing eNOS or denaturing eNOS. The Triton-induced membrane damage may lead to leakage of raw materials (both substrates such as L-arginine and cofactors such as BH4) necessary for NO generation through the plasma membrane 22. To investigate whether the TXL-induced protective effect is dependent on the activation of eNOS in endothelium, a Triton X-100 (0.125 %) bolus solution was used to abolish eNOS-mediated NO production 9,23. Triton-X 100 at a concentration of 0.25%, which is commonly used by other research groups 9,23, increased infarct size in our preliminary experiments. Therefore, a lower concentration of Triton-X 100, which had no impact on infarct size but significantly reduced NO production, was utilized in our study.

Infarct size was measured 1 h after reperfusion. The hearts were harvested and stored at -80°C overnight. The steps for TTC staining were the same as those described for measuring infarct size after *in vivo* I/R. ImageJ software (National Institutes of Health) was used to measure the IA and total cross-sectional heart area (TA). A percentage of TA (IA/TA) was used to express the infarct size.

### Measurement of nitric oxide production

Total NO production in cardiac tissues was determined by measuring the concentration of nitrate and nitrite, the stable metabolites of NO, by the modified Griess reaction method, according to the manufacturer's instructions (Beyotime Institute of Biotechnology, Shanghai, China). Optical density was measured at 540 nm and the amount of nitrite in the tissue lysate was calculated using sodium nitrite as a reference standard.

### Determination of CK-MB release in serum

Blood samples were collected from the abdominal aorta of rats using a 10 mL syringe 3 hours after reperfusion. Blood samples were collected and serum was separated by centrifugation and then kept at -80°C. The levels of CK-MB were measured using a commercially available Rat Elisa Assay kit with a microplate reader according to the manufacturer's instructions (Jiancheng Bioengineering Institute, Nanjing, China).

### Cell Culture and Treatments

Human CMs isolated from the ventricles of human adult hearts were purchased from PromoCell (Heidelberg, Germany). Cells were grown in a monolayer to 80% confluence and then sub-cultured using Ready-to-Use Myocyte Growth Medium (PromoCell). Experiments with human CMs were performed at passages three to seven.

Human CMECs were purchased from ScienCell Research Laboratories (San Diego, CA, USA) and cultured according to the manufacturer's instructions. Cells were grown in endothelial cell medium (ScienCell Research Laboratories) containing 5% fetal bovine serum (ScienCell Research Laboratories), 1% endothelial cell growth supplement (ScienCell Research Laboratories), and 1% penicillin/streptomycin at 37°C with 5% CO_2_, and then sub-cultured at a ratio of 1:3 when reaching 90% confluence. Cells in passages three to seven were used in our experiments.

Human cardiac fibroblasts were bought from ScienCell Research Laboratories. Human cardiac fibroblasts between passages 3-7 at 70-80% confluence were used for experiments. Human cardiac fibroblasts were cultured on poly-L-lysine coated culture plates in fibroblast medium (ScienCell) containing 10% FBS, growth supplements and penicillin/streptomycin, according to the manufacturer's instructions.

For co-culture experiments, 120,000 CMs were seeded into 6-well plate inserts with 0.4 μm pores (Corning, New York, USA) in normal culture medium. CMs were allowed to attach for 24 h before different treatments such as TXL pretreatment (400 μg/mL, 1 h) or siRNA transfection, and then the inserts were placed into 6-well plates previously plated with CMECs.

For H/R experiments, cells were washed with warm PBS twice and then exposed to different treatments in serum-free DMEM medium. Then cells were kept in an airtight and hypoxic GENbox jar fitted with a catalyst (BioMérieux, Marcy l'Etoile, France) to remove oxygen and induce 18 h hypoxia, as previously described 24. They were then placed under normal conditions for 2 h of reoxygenation. To ensure that the *in vitro* H/R model was successfully established, an anaerobic indicator dye (BioMérieux) was used to reflect the oxygen tension of the medium.

To investigate whether TXL protected CMECs via the eNOS pathway, CMECs were washed with phosphate buffered saline (PBS) and exposed to L-NNA (100 μM) 9 in serum-free DMEM for 30 min prior to hypoxia and throughout the H/R conditions in the absence or presence of TXL (800 μg/mL) 25***.***

For sEV inhibition, human CMs were treated in 6-well plate inserts (Corning) with a chemical sEV inhibitor, GW4869 26,27 (10μm) (Sigma, USA) for 24 h. Then, they were co-treated with TXL (400 μg/mL) 18 and GW4869 for 1 h prior to hypoxia, and then exposed to GW4869 alone when subjected to H/R. Human CMs were treated with DMSO as the vehicle control.

For all the *in vitro* experiments, at least 3 independent batches of CMECs, CMs or cardiac fibroblasts were used.

### Assessment of Morphological Changes

The chromatin dye Hoechst 33,342 was used to stain and assess cell nuclear condensation and fragmentation (Beyotime Institute of Biotechnology, China). Briefly, CMECs were fixed with paraformaldehyde (4%) for 30 min and then treated with 5 mg/mL Hoechst 33,342 for 30 min. Finally, stained cells were washed twice with PBS at room temperature and observed under a fluorescence microscope (Leica, Germany). Cells with fragmented and condensed nuclei were defined as dead cells.

### Determination of Cell Death by flow Cytometry

The percentage of dead cells was evaluated using the Annexin V-FITC/PI Kit (Becton, Dickinson and Company, USA), following the manufacturer's instructions. Briefly, CMECs, after experimental treatments, were collected and resuspended in 100 µl 1× binding buffer. The cell suspension was incubated with Annexin V and propidium iodide (PI) at room temperature in the dark for 15 min. Next, the binding buffer was mixed with another 400 µl 1× binding buffer, and CMECs were harvested and analyzed using the FACS Calibur System (Becton-Dickinson). Viable CMECs were considered annexin V^-^/propidium iodide (PI)^-^, early apoptotic CMECs as annexin V^+^/PI^-^ and late apoptotic CMECs and already dead CMECs as annexin V^+^/PI^+^. Cells of annexin V^+^/PI^-^ or annexin V^+^/PI^+^ were added together to calculate the proportion of dead CMECs.

### SEV isolation

SEVs were isolated by differential centrifugation described in detail elsewhere 28. Briefly, to isolate sEVs derived from CMs that were untreated, pretreated with TXL, or treated with lincRNA-ROR siRNA or conditioned medium from cells pretreated with TXL pretreatment (1 h) and then stimulated by H/R (18 h/2 h) were harvested and centrifuged. Cells and debris were removed by differential centrifugation at 300 ×g (10 min) and 2000 ×g (20 min), respectively. Microvesicles were precipitated by centrifuging at 13,500 ×g (30 min) and discarded. Supernatants were then ultra-centrifuged at 120,000 ×g (70 min) to acquire a raw sEV pellet. The pellet was then rinsed with PBS followed by a second ultracentrifugation (120,000 ×g, 70 min). The sEV pellet was resuspended in PBS and stored at -80°C for the corresponding experiments. The whole procedure was undertaken under sterile conditions.

### Transmission electron microscopy (TEM)

SEVs diluted in PBS were adsorbed to a 200-mesh formvar/carbon coated electron microscopy grid (Electron Microscopy Sciences, PA, USA) and incubated at room temperature for 5 min. Next, excessive PBS was removed and 0.75% uranyl acetate dihydrate was used to stain sEVs for 5 min, followed by rinsing the samples with PBS three times. After the excess liquid was removed, the grids were observed by TEM and images were recorded with a camera.

### Fluorescence confocal microscopy for sEV uptake

To determine how fast sEVs were taken up by CMECs *in vitro*, isolated sEVs were labeled with fluorescent dye PKH26 using a Red Fluorescent Cell Linker Kit (Sigma-Aldrich) according to the manufacturer's instructions and rinsed with PBS followed by ultra-centrifuging twice to remove redundant dye. PKH26-labeled sEVs were incubated with CMECs for 1 or 18 h. CMECs were then rinsed twice with PBS, fixed with 4% paraformaldehyde for 15 min, rinsed twice with PBS and permeabilized with 0.1% Triton X-100 for 5 min. Thereafter, cells were blocked with 1% goat serum for 40 min and stained with phalloidine and DAPI at room temperature. Uptake of PKH26-labeled sEVs by CMECs was observed by confocal microscopy.

### Real-Time RT-PCR

Total RNA from CMECs, human CMs or sEVs was extracted using Trizol, according to the manufacturer's protocol (Invitrogen). linc-ROR and GAPDH mRNAs were reverse transcribed to cDNA using an iScript cDNA Synthesis Kit (Bio-Rad, Hercules, CA, USA) and then quantified by quantitative real-time RT-PCR with SYBR Green PCR Master Mix. MicroRNAs were reverse transcribed using a miScript II RT Kit (Qiagen, Valencia, CA, USA) and quantified by quantitative real-time RT-PCR using a miScript SYBR green PCR kit (Qiagen). qRT-PCR was performed on an ABI 7500 thermocycler (Applied Biosystems) for 40 cycles. The following PCR primers were used: GAPDH forward, 5′-GAAGGTGAAGGTCGGAGTCA-3′; reverse, 5′-GGAAGATGGTGATGGGATTTC-3′; linc-ROR forward, 5′-AGGAAGCCTGAGAGTTGGC-3′; reverse, 5′-CTCAGTGGGGAAGACTCCAG-3′. Specific primers for microRNAs (miR-497-5p, miR-145-5p, miR-128-3p, and U6) were purchased from Qiagen. The linc-ROR from CMECs or human CMs was quantified using the 2^(-ΔΔCT)^ relative quantification method, using GAPDH as an internal control. Linc-ROR in sEVs was quantified using the 2^(-ΔΔCT)^ relative quantification method, using ce-miR-39-3p as an external control, as previously described 29, 30. MicroRNA levels were quantified by the 2^(-ΔΔCT)^ relative quantification method, using U6 as an internal control.

### Western Blotting

Proteins were extracted from CMECs, sEVs or myocardial tissue samples. The BCA assay (Beyotime Institute of Biotechnology, China) was used to measure protein concentrations. To assess protein levels in these samples, lysates containing 25μg protein were separated on NuPage 4%-12% Bis-Tris Gels (Novex, Life Technologies) and protein bands were transferred to nitrocellulose membranes using a dry electroblotting apparatus (Invitrogen). Then 5% non-fat dry milk was utilized to block the membranes. Membranes were incubated in solutions of primary antibodies overnight at 4°C. The primary antibodies used included eNOS (#32027; 1:1000) and phospho-eNOS (Ser 1177) (#9570; 1:1000), Calnexin (#2679S; 1:1000), all from Cell Signaling Technology. Other primary antibodies used were p70s6k1(ab32529; 1:5,000), phosphop70s6k1 (Thr389) (ab2571; 1:250), CD81(ab109201; 1:2000) and CD63 (ab134045; 1:2000), all from Abcam. After being incubated with primary antibodies, membranes were rinsed three times and incubated with appropriate secondary antibodies (1:5,000; Zhongshanjinqiao, China) on the second day. Then stained bands were visualized with a Chemiluminescence Detection Kit (Pierce) after washing membranes three times. Protein signals were normalized to GAPDH (ab181602; primary antibody at 1:5,000; Abcam, Cambridge, UK). ImageJ software was used to analyze the densitometry.

### Cell Transfection

Small interfering RNA (siRNA) oligonucleotides against p70s6k1 (Santa Cruz Biotechnology), nonspecific control siRNA oligonucleotide (Santa Cruz Biotechnology), mimics of miR-145-5p (Thermo Fisher Scientific, Inc., Waltham, MA, USA) or the microRNA negative control (Thermo Fisher Scientific, Inc.) were transfected into CMECs using Lipofectamine™ 2000 (Thermo Fisher Scientific, Inc.) in Opti-MEM Reduced Serum Medium (Thermo Fisher Scientific, Inc.). siRNA against linc-ROR (Genepharma, Shanghai, China) and nonspecific control siRNA oligonucleotides (Genepharma, Shanghai, China) were transfected into human CMs with the above-mentioned reagents. Cells were used in the following experiments after 48 h transfection. The siRNA sequence for linc-ROR was 5'-GGAGAGGAAGCCTGAGAGT-3'. The other proprietary sequences of siRNAs and microRNAs were not divulged by the companies.

### Statistical Analysis

Statistical analysis was conducted in the GraphPad Prism 8.0.1. Quantitative data are displayed as the means ± standard error of the mean (SEM). Continuous variables between two groups were compared by the Student *t*-test. In the experiment of microcirculatory perfusion, two-way analysis of variance (ANOVA) was used for data comparisons. Comparison in other experiments with more than two groups was performed by one-way ANOVA. Following either one-way or two-way ANOVA, post-hoc analysis by Tukey's test utilized for multiple comparisons. P < 0.05 was considered to show statistical significance.

## Results

### Indirect activation of eNOS in CMECs can induce cardioprotection after I/R

**Figure 1:**
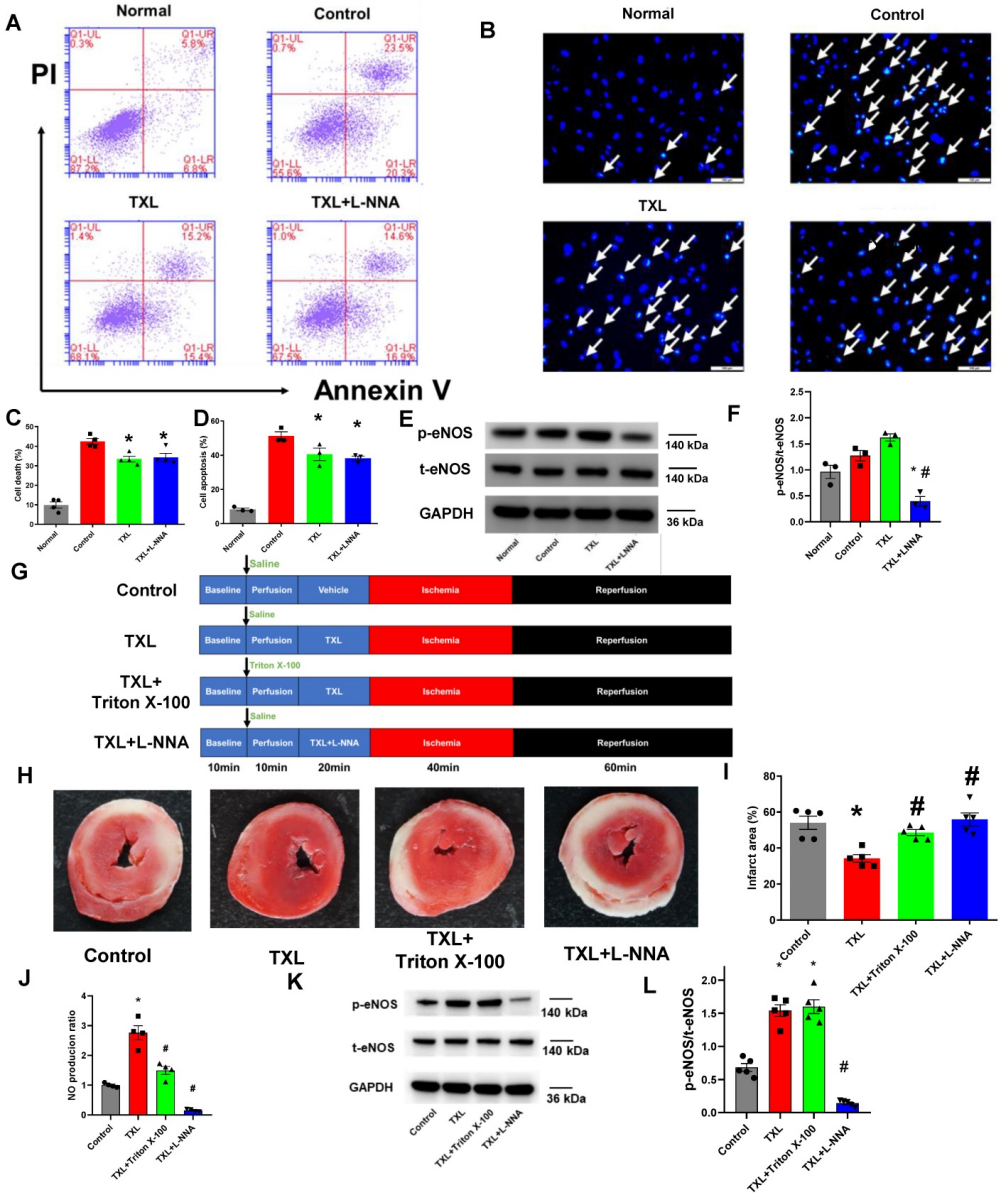
Indirect activation of eNOSin CMECs can induce cardioprotectionafter I/R.A) Representative scatter diagram of the death of CMECs in flow cytometry assay (n=4). B) Representative images of Hoechst 33342 nucleic acid staining for CMECs; white arrows point to dead cells (Bar=100 μm, n=3). C-D) Quantitative analysis for dead cells in flow cytometry assay and Hoechst staining, respectively. E-F) The p-eNOSand t-eNOSwere detected by Western Blot for the *in vitro* experiments (n=3). G) A schematic representation of the experimental protocol for the Langendorffmodel. H) Representative images of TTC staining in each group (*n* = 5, 5-6 slices per heart). The red-stained areas indicate viable tissue, and the pale white areas indicate infarct tissue. I) Quantitative analysis for infarct size in each group. J) NO production in cardiac tissues for each group (Griess reaction method (n=5). K-L) The p-eNOSand t-eNOSwere detected by Western Blot for the *ex-vivo*hearts (n=5). *P<0.05 vs. Control; #P <0.05 vs. TXL. Control: H/R or I/R; All data are mean ±SEM. Statistical analysis was performed with one-way ANOVA followed by Tukey's test.

Activation of eNOS is often required by beneficial agents to confer protection on endothelial cells under pathological conditions 31, 32. For instance, the anti-apoptotic effect of sphingosine 1-phosphate on endothelial cells stressed with ethanol is dependent on the increased activity of this enzyme 31. As we previously showed that TXL could protect CMECs from H/R-induced injury *in vitro* 25, and more importantly, the infarct-sparing effect of TXL in the *in-vivo* model of I/R required the activation of eNOS 16, 17, we first hypothesized that TXL was able to directly activate the eNOS pathway in CMECs, leading to the reduction of H/R-induced CMEC death. To test this hypothesis, we exposed CMECs to a model of acute simulated ischemia/reperfusion injury (18 h hypoxia followed by 2 h reoxygenation), as previously described 18. As shown in **Figure S1A-B**, TXL had few effect (10.45 ± 0.92% vs 9.10 ± 1.01% in the Normal group, P > 0.05) on the viability of CMECs under normal conditions. Consistent with our previous research, TXL slightly inhibited the cell death of CMECs under the H/R conditions (33.43 ± 1.45% vs 42.53 ± 1.49% in the H/R group, P < 0.05) (**Figure 1A, C**), as detected by flow cytometry assay. Surprisingly, L-NNA, an inhibitor of eNOS, failed to inhibit the protective effect of TXL (34.30 ± 2.11% vs 33.43 ± 1.45% in the TXL group, P > 0.05) (**Figure 1A, C**), despite its efficient inhibition of eNOS activity (0.40 ± 0.09 vs 1.62 ± 0.07 in the TXL group, P < 0.05) (**Figure 1E-F**). The above findings were further confirmed by Hoechst 33342 staining (38.16 ± 1.44% vs 40.6 ± 3.68% in the TXL group, P > 0.05) **(Figure 1B, D)** and the CCK-8 assay (81.05 ± 1.43% vs 78.54 ± 1.51% in the TXL group, P > 0.05) (**Figure S2).** Importantly, eNOS was not activated by TXL in these conditions (**Figure 1E-F**). These results suggested that the protective effect of TXL on I/R injury is not through a direct activation of the eNOS pathway in CMECs.

Our earlier work demonstrated a key role for eNOS in the cardioprotective effects of TXL 16, 17. Therefore, we next asked if the eNOS activation observed in TXL-treated hearts was from CMECs and more importantly, if this CMEC derived eNOS was required for the cardioprotective effects of TXL. To address this question, we utilized the Langendorff model and exposed rat hearts to I/R injury (**Figure 1G**). As expected and shown in **Figure 1H-I**, TXL treatment significantly decreased the infarct size after I/R (34.14 ± 2.14% vs 54.02 ± 3.69% in the Control group, P < 0.05). Treatment with L-NNA, a competitive inhibitor of nitric oxide synthase, was effective in repressing the phosphorylation of eNOS (0.14 ± 0.02 vs 1.54 ± 0.09 in the TXL group, P < 0.05) (**Figure 1K-L**). Repression of eNOS activity in this manner completely abrogated the cardioprotective benefits of TXL treatment (55.84 ± 3.65% vs 34.14 ± 2.14% in the TXL group, P < 0.05) (**Figure 1H-I**). As shown in **Figure S3**, compared with the Control group, TXL group did not display an increase in p-nNOS/t-nNOS or the expression of iNOS, which ruled out the possibility that L-NNA took effects in our *ex-vivo* study by inhibiting the activity of nNOS or iNOS. In addition, we found that L-NNA treatment alone had no significant impact on infarct size (**Figure S4A-B**). Previously, low levels of Triton-X 100 pretreatment has been shown to be effective in abolishing coronary endothelial eNOS-mediated NO production with negligible effects on myocardial function and infarct size 22, 23. We therefore pretreated the hearts with Triton-X 100 under these conditions and examined the impact on infarct size and TXL cardioprotection. Triton-X 100 pretreatment alone did not significantly change the infarct size after I/R (57.13 ± 4.26% vs 54.02 ± 3.69% in the Control group, P > 0.05, **Figure S4A-B**). In stark contrast, Triton-X 100 pretreatment dramatically inhibited the cardioprotective effects of TXL (48.54 ± 1.67% vs 34.14 ± 2.14% in the TXL group, P < 0.05) (**Figure 1H-I**). As shown in **Figure 1J,** Triton-X 100 pretreatment significantly reduced the level of NO in cardiac tissues (1.50 ± 0.13 vs 2.76 ± 0.24 in the TXL group, P < 0.05), indicating that it efficiently disrupted the function of eNOS in the coronary endothelium. Importantly, as shown in **Figure 1K-L**, Triton-X 100 inhibits NO without affecting the increased phosphorylation of eNOS induced by TXL (1.60 ± 0.10 vs 1.54 ± 0.09 in the TXL group, P > 0.05), demonstrating that the Triton-X treatment is not merely disrupting TXL uptake into cells. The correlation of decreased cardioprotection with decreased NO production are in agreement with our previous studies that TXL attenuated I/R injury in an eNOS-dependent pathway* in vivo* 16,17. However, these data further suggest that the cardioprotection induced by TXL in *ex vivo* hearts were dependent on the activation of eNOS in cardiac endothelial cells. Taken together, the data from these experiments suggested that cardioprotection, induced by certain agents such as TXL, can result from the indirect activation of eNOS in CMECs during I/R.

### Evidence of eNOS activation in CMECs through a cardiomyocyte-dependent paracrine pathway

**Figure 2:**
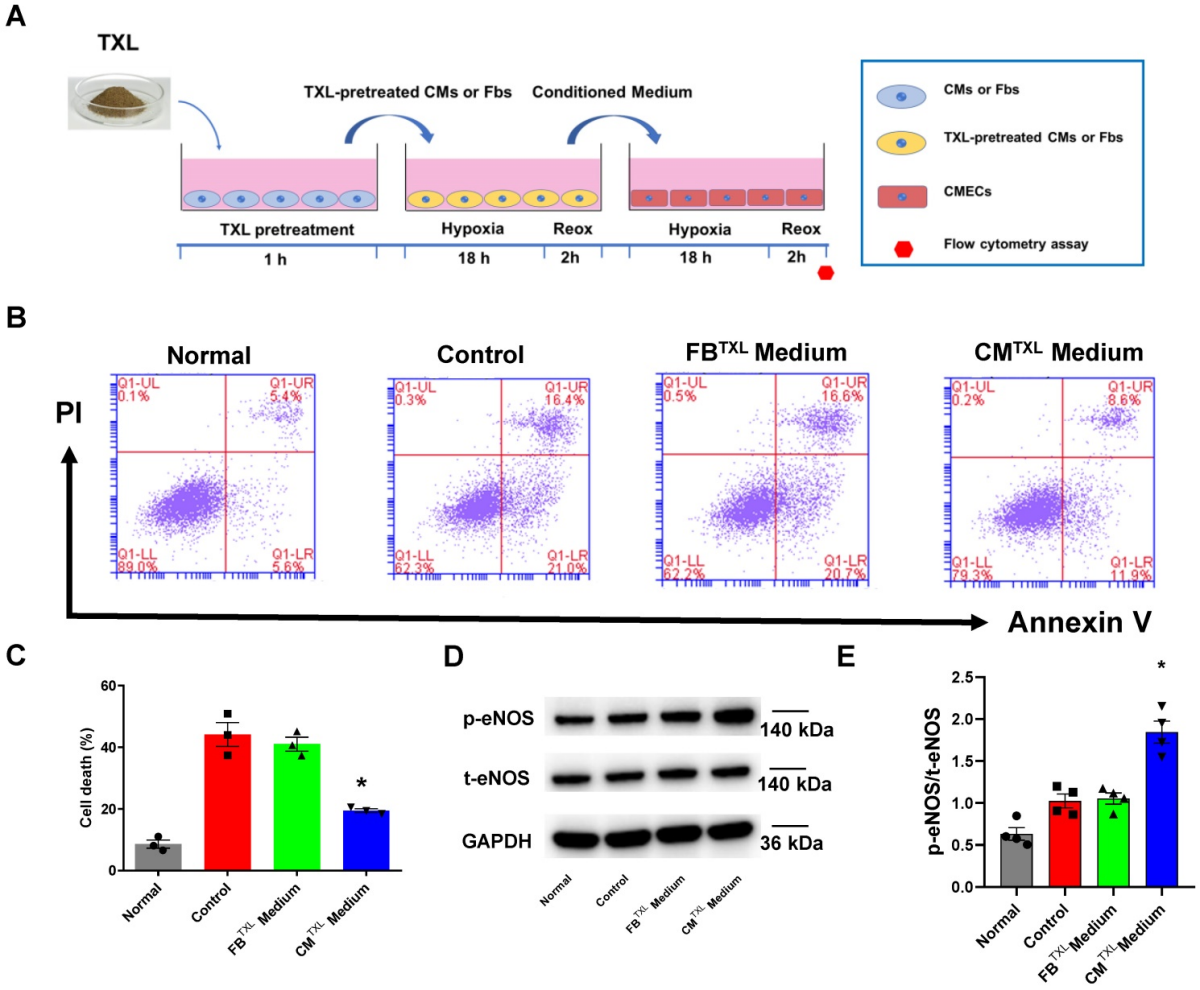
Evidence of eNOSactivation in CMECs through a cardiomyocyte-dependent paracrine pathway. A) Schematic of the experimental protocol for investigating the effects of conditioned medium of CMs or Fbson CMECs. B-C) Dead CMECs in flow cytometry assay (n=3). D-E) The p-eNOSand t-eNOSwere detected by Western Blot (n=4). *P<0.05 vs. Control. CM: cardiomyocyte; CM^TXL^: TXL-pretreated cardiomyocyte; Fb: fibroblast; Fb^TXL^: TXL-pretreated fibroblast; Reox: reoxygenation. All data are mean ±SEM. Statistical analysis was performed with one-way ANOVA followed by Tukey's test.

Our data suggests that the activation of endothelial eNOS is required by TXL to exert its infarct-sparing effect in the *ex vivo* model **(Figure 1H-I)**. In addition, our *in vitro* experiments demonstrated that TXL did not directly activate eNOS in endothelial cells (**Figure 1E-F**). Furthermore, our *ex vivo* results **(Figure 1H-I)** rule out the possibility from our earlier *in vivo* studies that TXL conferred its cardioprotective effect via a systemic effect 16, 17. Taken together, we hypothesized that TXL promotes crosstalk between endothelial and non-endothelial cells in hearts that promotes cell survival during I/R. Because fibroblasts (approximately 62.6% in rats, and approximately 26.1% in mice) and cardiomyocytes (approximately 26.4% in rats, and approximately 55.9% in mice) are the other two main types of cells in hearts 33, we used their conditioned medium to treat CMECs under H/R conditions **(Figure 2A)**. In this experiment, we pretreated fibroblasts and cardiomyocytes with TXL for 1 h and then subjected them to H/R, after which their conditioned medium was collected (Fb^TXL^ medium and CM^TXL^ medium) and used to treat CMECs when exposed to H/R. As shown in **Figure 2B-C**, compared with the Control group, the group with CM^TXL^ medium had significantly fewer numbers of dead cells (19.43 ± 0.61% vs 44.1 ± 3.90% in the Control group, P < 0.05), while no difference was observed between the Control and Fb^TXL^ medium groups (41.00 ± 2.26% vs 44.1 ± 3.90% in the Control group, P > 0.05). These findings were confirmed in the CCK-8 assay, showing that CM^TXL^ medium, rather than Fb^TXL^ medium, could protect CMECs from H/R-induced injury **(Figure S5)**. Of note, conditioned medium from Fb or CM without TXL pretreatment had few effects on the cell death of CMECs (44.1 ± 3.90% vs 42.77 ± 3.09% in the Fb medium group and 39.63 ± 2.96% in the CM medium group, P > 0.05)** (Figure S6A-B)** during H/R, supporting the model in which TXL promotes crosstalk between these cell types to increase survival. Furthermore, CM^TXL^ medium significantly increased the phosphorylation of eNOS in CMECs (1.84 ± 0.13 vs 1.03 ± 0.08 in the Control group, P < 0.05) **(Figure 2D-E)**, suggesting that TXL treatment not only induces CMs to release a paracrine factor increasing endothelial cell survival after I/R, but also stimulates eNOS activity in these cells leading to an increase in cardioprotection.

### CM-derived sEVs mediate the eNOS activation in CMECs during H/R in a trans-well co-culture system

**Figure 3:**
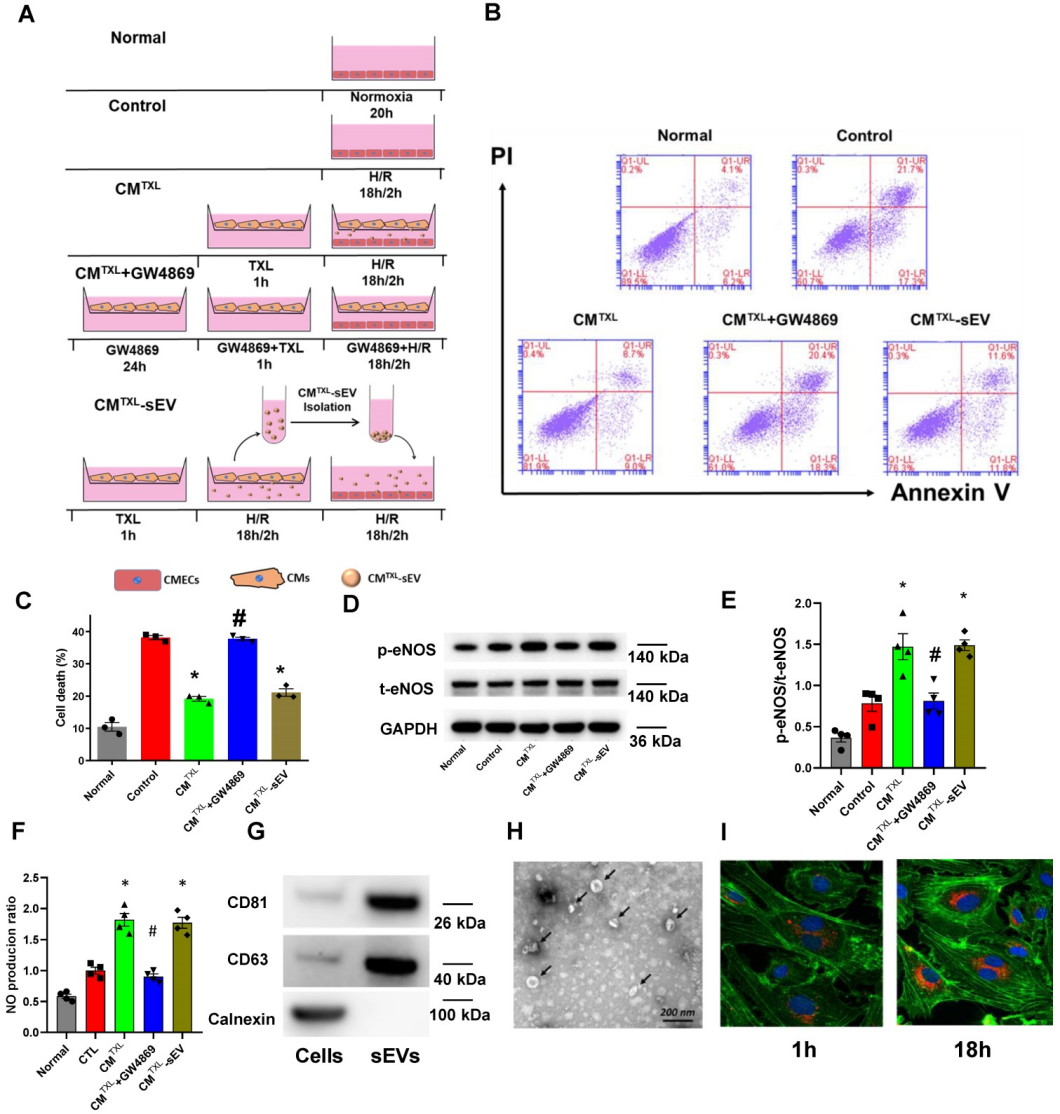
CM-derived sEVsmediate the eNOSactivation in CMECs during H/R in a trans-well co-culture system.A) Schematic of the protocol for co-culture experiments. B-C) Dead CMECs in flow cytometry assay (n=3). D-E) The p-eNOSand t-eNOSwere detected by Western Blot (n=4). F) NO production in CMECs for each group (Griess reaction method, n=4). G) Western blots confirmed the presence of sEVmarker in sEVsderived from TXL-pretreated CM after H/R (n=4). H) Electron micrographs of purified sEVsfrom TXL-pretreated CM after H/R. (Bar: 200 nm; n=3). I) Representative confocal images showing that red fluorescence dye PKH26 labelled sEVsfrom TXL-pretreated CM after H/R were endocytosed by CMECs after 1 hour and 18 hours incubation. *P<0.05 vs. Control; #P <0.05 vs. CM^TXL^. sEVs: small extracellular vesicles; CM: cardiomyocyte; CM^TXL^: TXL-pretreated cardiomyocyte; CM^TXL^-sEV: sEVsderived from CM^TXL^. All data are mean ±SEM. Statistical analysis was performed with one-way ANOVA followed by Tukey's test.

Our data suggest that treatment of CMs with TXL results in the release of a factor(s) that decreases endothelial cell death during I/R. SEVs, previously named as exosomes 34, are nanosized particles released into the extracellular space by various cell types. They contain substances such as proteins, lipids and nucleic acids (mRNA, long non-coding RNA, etc) and have an important role in cell-to-cell communication 35. Close contact between CMs and CMECs suggests that sEVs can mediate the crosstalk between these two types of cells. Indeed, one study demonstrated that cardiomyocytes mediated anti-angiogenesis via the sEV transfer of miR-320 into endothelial cells in type 2 diabetic rats 36. Furthermore, sEVs released from endothelial cells have been suggested, in some cases, to protect cardiomyocytes from H/R-induced injury 8. Taken together, we hypothesized that TXL-pretreated cardiomyocytes protect endothelial cells exposed to H/R by releasing sEVs. To test this hypothesis, a trans-well co-culture system was introduced into our experiments **(Figure 3A)**. As expected, being co-cultured with CM^TXL^, instead of CM (37.47 ± 0.64% in the CM group vs 40.03 ± 2.06% in the Control group, P > 0.05)** (Figure S7),** inhibited the cell death of CMECs during H/R (19.17 ± 0.74% vs 38.13 ± 0.63% in the Control group, P < 0.05)** (Figure 3B-C)**, validating the results of the above experiments **(Figure 2B-C)**. However, this beneficial effect was totally abolished when GW4869, an sEV release inhibitor, was used to treat CM^TXL^ (37.73 ± 0.55% vs 38.13 ± 0.63% in the Control group, P > 0.05)** (Figure 3B-C)** , suggesting a requirement for secreted sEVs in the CM^TXL^ mediated protection of CMECs during H/R. GW4869 alone did not affect the death of CMECs (37.87 ± 0.59% vs 38.13 ± 0.63% in the Control group, P > 0.05) (**Figure S8A-B**) or CM (21.53 ± 1.25% in the CM^TXL^ group vs 20.23 ± 1.11% in the CM^TXL^+GW4869 group, P > 0.05) (**Figure S9A-B**) under H/R conditions. sEVs released from H/R-treated CM^TXL^ (CM^TXL^-sEVs) were also isolated and incubated with CMECs. CM^TXL^-sEVs were able to alleviate the H/R-induced injury of CMECs (21.10 ± 1.15% vs 38.13 ± 0.63% in the Control group, P < 0.05)** (Figure 3B-C)**, while CM-sEV **(Figure S7)** or CM^TXL^-medium depleted of sEVs **(Figure S10)** did not affect the death of CMECs under H/R. Additionally, sEVs derived from H/R-treated NRVM^TXL^ (NRVM^TXL^-sEVs; NRVM refers to neonatal rat ventricular myocyte) were isolated and used to treat rat aortic endothelial cells. Consistently, NRVM^TXL^-sEVs significantly inhibited the cell death of rat aortic endothelial cells exposed to H/R (28.27 ± 1.13% vs 44.90 ± 2.38% in the Control group, P < 0.05)** (Figure S11A-B)**.

While TXL did not induce direct CMEC protection against H/R through eNOS activation, this activation of eNOS by TXL is critical to the cardio-protective benefits of the treatment. We therefore investigated the requirement of eNOS activity in the CM^TXL^ derived protection of CMECs to H/R. Surprisingly, under these conditions, eNOS was necessary for the protective effect of CM^TXL^-sEVs on CMECs to H/R **(Figure S12A-B)**. As shown in **Figure 3D-E**, compared with the Control group, CMECs in the CM^TXL^ group had a significantly higher ratio of p-eNOS/t-eNOS (1.47 ± 0.16 vs 0.78 ± 0.10 in the Control group, P < 0.05). This trend was reversed when GW4869 was introduced to inhibit the release of sEVs from CM^TXL^ (0.81 ± 0.10 vs 0.78 ± 0.10 in the Control group, P > 0.05) **(Figure 3D-E)**. The treatment of CMECs with CM^TXL^-sEVs also facilitates the phosphorylation of eNOS compared with the Control group (1.49 ± 0.07 vs 0.78 ± 0.10 in the Control group, P < 0.05) (**Figure 3D-E**). Our further experiments (**Figure 3F**) showed that CMECs in the CM^TXL^ group had more NO production (1.82 ± 0.10 vs 1.00 ± 0.05 in the Control group, P < 0.05), compared with the Control group. However, this effect could be offset by GW4869 (0.90 ± 0.04 vs 1.00 ± 0.05 in the Control group, P < 0.05) (**Figure 3F**). Additionally, treating CMECs with CM^TXL^-sEVs also augmented the production of NO (1.77 ± 0.09 vs 1.00 ± 0.05 in the Control group, P < 0.05) (**Figure 3F**), compared with the Control group. These data show that CM-derived sEVs are necessary and sufficient for the CM^TXL^ activation of eNOS in the CMECs.

To further support the above findings, western blotting, transmission electron microscopy (TEM) and nanoparticle tracking analysis (NTA) were used to characterize sEVs derived from CM^TXL^. Western blot analyses demonstrated that CM^TXL^-sEVs had a high expression of the sEV specific markers, CD81 and CD63, while the marker of cell lysate, Calnexin, could not be detected **(Figure 3G)**. TXL pretreatment had no impact on CM's ability to release sEVs, as demonstrated by the amount of these markers (**Figure S13A**) in the conditioned medium from CM or CM^TXL^ and shown in NTA (**Figure S13B**). CM^TXL^ pretreated with GW4869 secreted much less sEVs compared with the group without GW4869 pretreatment, as shown by the dramatical reduction of CD81 and CD63 in the conditioned medium of CM^TXL^ (**Figure S14A**), suggesting the efficacy of GW4869 in our conditions to inhibit sEV release. TEM imaging analysis showed that CM^TXL^-sEVs had a cup-shaped structure and were approximately 100 nm in diameter **(Figure 3H)**. To assess the internalization of sEVs by CMECs, CM^TXL^-sEVs were pre-labeled with PKH26 (a red fluorescent dye that stains the cell membrane) and cultured with CMECs. Confocal images showed that CMECs had internalized many CM^TXL^-sEVs 1 h after incubation and had taken up a significantly higher amount of stained CM^TXL^-sEVs by 18 h **(Figure 3I)**, indicating CMECs internalize sEVs rapidly. PKH26 dye was also used to label conditioned medium from CM^TXL^ pretreated with GW4869, and there was no transfer of dye into CMECs, supporting the inhibition efficacy of GW4969 again (**Figure S14B**).

### The improvement of cardiac microcirculation and infarct-sparing effect induced by TXL is mediated by sEVs

**Figure 4:**
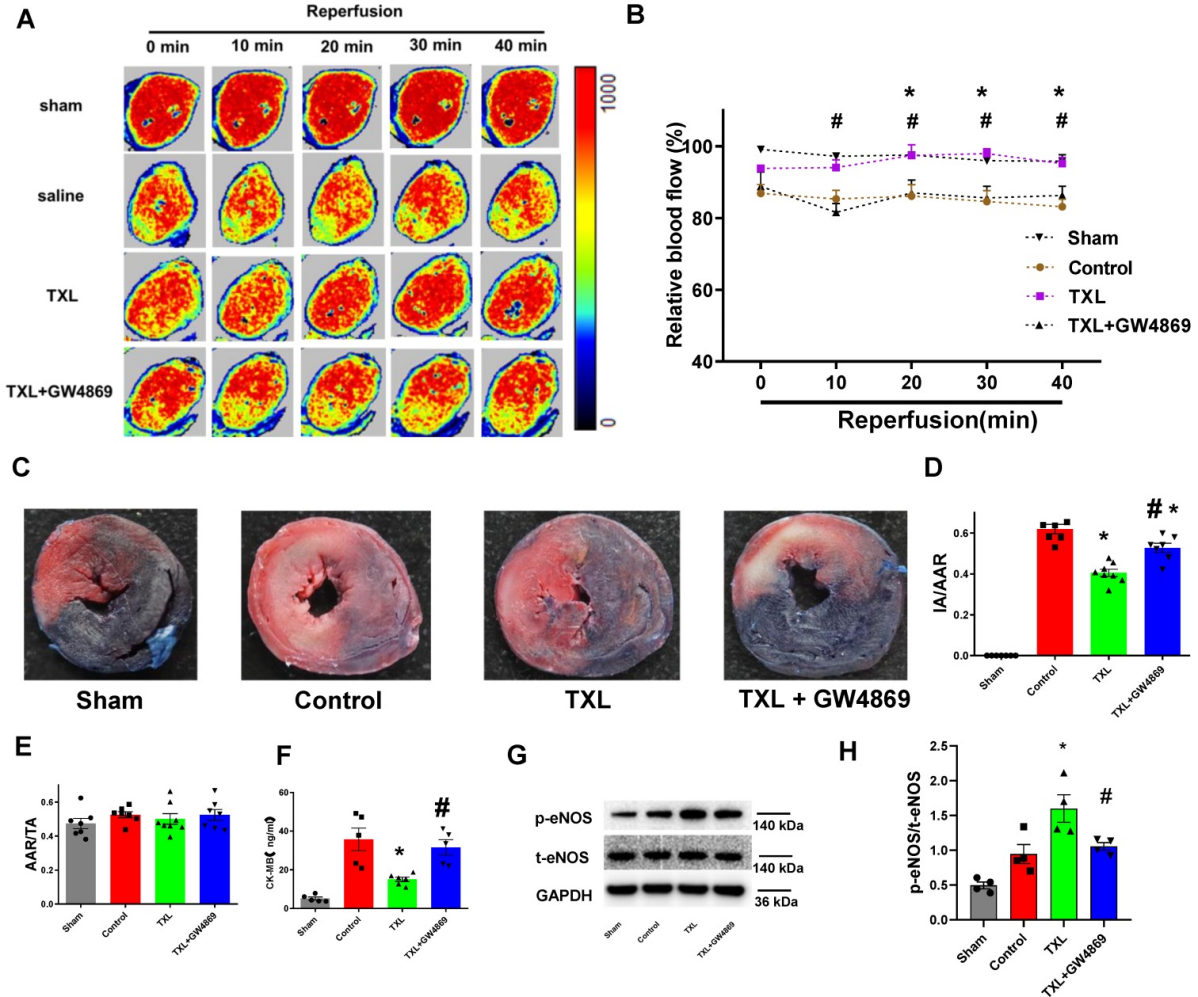
The improvement of cardiac microcirculation and infarct-sparing effect induced by TXL is mediated by sEVs.A-B) Representative perfusion images for myocardial microcirculatory perfusion (percentage relative to baseline) measured by laser Doppler flow during myocardial I/R (n=6-8), and the statistical graph were shown, respectively. C) Representative images for myocardial infraction size assessed by Evans blue/TTC double staining (n=7-8). D-E) Quantitative analysis for infarct size and area at risk after I/R, respectively. F) Serum CK-MB activity in each group (n=5-6). G-H) The p-eNOSand t-eNOSwere detected by Western Blot (n=4). * P<0.05 TXL vs. Control; # P <0.05 TXL + GW4869 vs TXL. AAR: area at risk; IA: infarct area; TA: total area. All data are mean ±SEM. Statistical analysis was performed with two-way (microcirculatory perfusion) or one-way (other experiments) ANOVA followed by Tukey's test.

To investigate if TXL could reduce the death of CMECs during I/R and thus improve cardiac microcirculation, we then used laser Doppler flowmetry to record cardiac microvascular perfusion after the release of coronary artery ligation. As hypothesized, the TXL group displayed a significant improvement in cardiac microcirculation after I/R, compared with the control group (**Figure 4A-B**).

To examine the requirement of sEVs in the *in vivo* protective effect induced by TXL, GW4869 was used to inhibit the secretion of sEVs *in vivo*. As shown in **Figure 4A-B,** GW4869 could offset the protective effects of TXL on cardiac microvascular perfusion.

Further experiments demonstrated that, the TXL group had a significantly decreased myocardial infarct size after I/R compared with the control group (40.51 ± 1.75% vs 61.89 ± 2.29% in the Control group, P < 0.05) (**Figure 4C-E**) , which was consistent with our published research 16-18. However, GW4869 pretreatment reduced the beneficial effect of TXL (52.64 ± 2.27% vs 61.89 ± 2.29% in the Control group, P > 0.05) **(Figure 4C-E)**. GW4869 did not on its own have any impact on infarct size under these conditions (61.73 ± 2.49% vs 59.57 ± 2.22% in the Control group, P > 0.05) **(Figure S15A-C)**. Serum CK-MB levels, a marker of cardiac cell death, was reduced in the TXL treated group compared to the untreated control after I/R (15.06 ± 1.16% vs 35.69 ± 5.89% in the Control group, P < 0.05) **(Figure 4F)**. Consistent with the infarct size, no difference in serum CK-MB levels was observed between the control and TXL+GW4869 groups (31.59 ± 3.91% vs 35.69 ± 5.89% in the Control group, P < 0.05) **(Figure 4F)**. And compared with the control group, the phosphorylation of eNOS was statistically increased in the TXL treated group (1.60 ± 0.20 vs 0.95 ± 0.14 in the Control group, P < 0.05) **(Figure 4G-H)**; however, this effect was counteracted by GW4869 (1.05 ± 0.05 vs 0.95 ± 0.14 in the Control group, P > 0.05) **(Figure 4G-H)**. We then isolated the adult rat CMECs after myocardial I/R **(Figure S16A-B)**, demonstrating that CMECs in the TXL-pretreated hearts had increased eNOS activity (1.73 ± 0.24% vs 0.81 ± 0.12% in the Control group, P < 0.05), which was offset by the treatment of GW4869 (0.80 ± 0.18% vs 0.81 ± 0.12% in the Control group, P > 0.05). These data support a model by which TXL-induced sEVs drive crosstalk between CMs and endothelial cells increasing eNOS production and cell survival after I/R.

### CM-derived sEVs mediate the eNOS activation in CMECs through the p70s6k1 pathway

**Figure 5:**
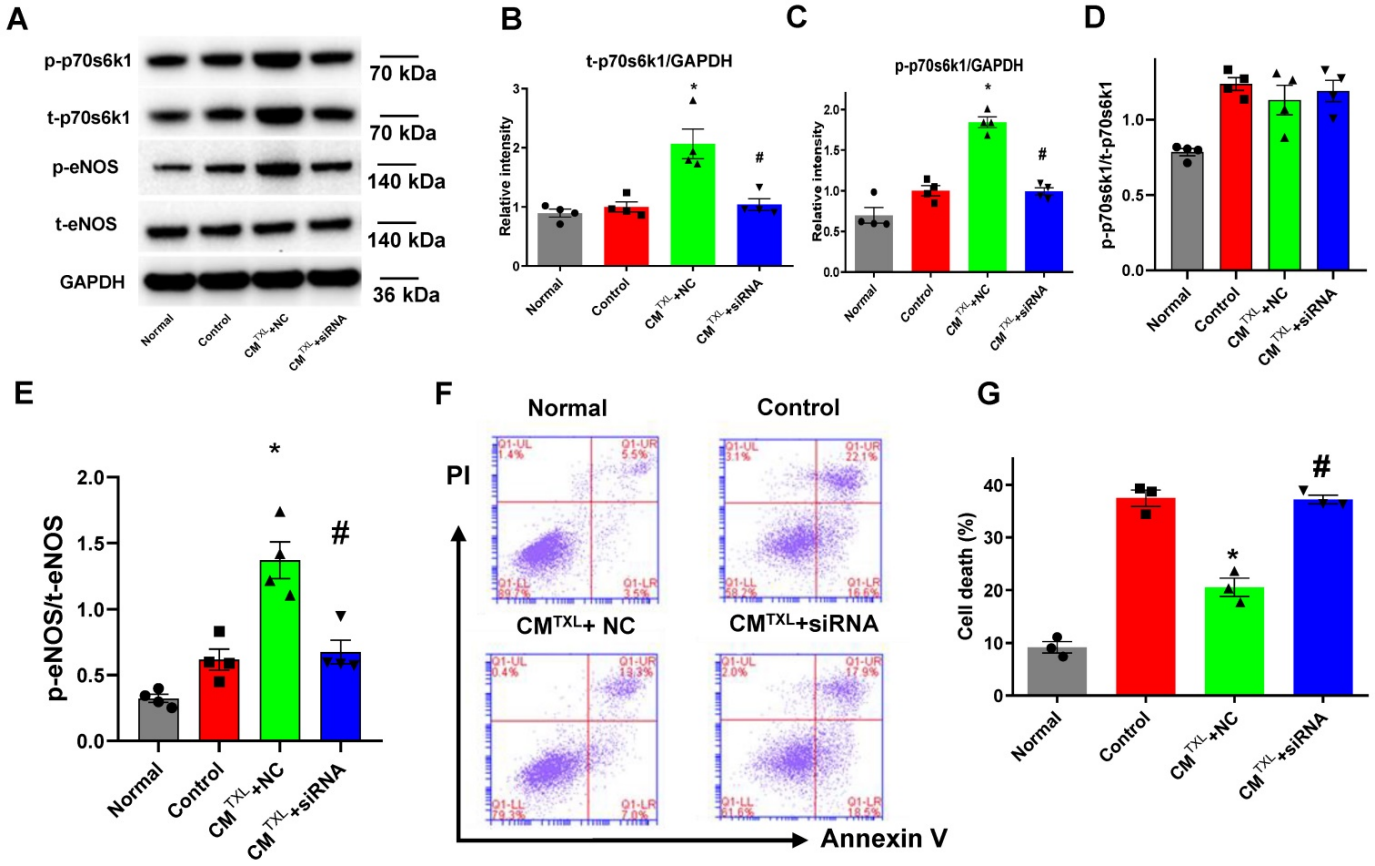
CM-derived sEVsmediate the eNOSactivation in CMECs through the p70s6k1 pathway. A-E) The levels of p-70s6k1, t-p70s6k1, p-eNOSand t-eNOSwere detected by Western Blot (n=4). F-G) Dead CMECs in flow cytometry assay (n=3). * P<0.05 vs. Control; # P <0.05 vs CM^TXL^+NC. sEVs: small extracellular vesicles; CM: cardiomyocyte; CM^TXL^: TXL-pretreated CMs; CM^TXL^+siRNA group: CMECs were transfected with p70s6k1 siRNA before being co-cultured with CM^TXL^ and then exposed to H/R; CM^TXL^+NC group: CMECs were transfected with negative control of siRNA before being co-cultured with CM^TXL^ and then exposed to H/R. All data are mean ±SEM. Statistical analysis was performed with one-way ANOVA followed by Tukey's test.

Previous studies have shown that cysteine-rich, angiogenic inducer 61 or neuropeptide Y activate the eNOS pathway by increasing the phosphorylation of p70s6k 37,38. Furthermore, our recent study reported that TXL attenuated I/R injury by upregulating the steady-state level of p70s6k1 and increasing p-p70s6k1 18. Consequently, we hypothesized that CM^TXL^-sEVs protect CMECs from H/R injury by activating the p70s6k1/eNOS pathway. As shown in **Figure 5A-D**, compared with the Control group, TXL^TXL^+NC group displayed higher levels of both t-p70s6k1 (2.07 ± 0.25 in CM^TXL^ + NC group vs 1.00 ± 0.08 in the Control group, P < 0.05) and p-p70s6k1 (1.84 ± 0.07 in CM^TXL^ + NC group vs 1.00 ± 0.06 in the Control group, P < 0.05), with no significant difference in the ratio of p-p70s6k1/t-p70s6k1 (1.13 ± 0.10 in CM^TXL^ + NC group vs 1.24 ± 0.04 in the Control group, P > 0.05) being found between these two groups. Consistently, similar results could be observed in the adult rat CMECs isolated after myocardial I/R (**Figure S16A, C-E**). These data indicated that the increased p-p70s6k1 level in the CM^TXL^+NC group was dependent on the augmentation of its steady-state level. In addition, transfection of siRNA against p70s6k1 into CMECs reversed the increasing trend of t-p70s6k1 levels (1.04 ± 0.10 in CM^TXL^ + siRNA group vs 1.00 ± 0.08 in the Control group, P > 0.05) **(Figure 5A-B)** induced by co-culture with CM^TXL^ under H/R conditions; thus, the level of p-p70s6k1 (0.99 ± 0.04 in CM^TXL^ + siRNA group vs 1.00 ± 0.06 in the Control group, P > 0.05) **(Figure 5A, C)** in CMECs was unchanged when co-cultured with CM^TXL^. As hypothesized, the decreased level of p-p70s6k1, caused by the reduction in the expression of t-p70s6k1, inhibited the activation of eNOS (0.67 ± 0.09 in CM^TXL^ + siRNA group vs 0.62 ± 0.08 in the Control group, P > 0.05) **(Figure 5A, E)** in CMECs induced by co-culture with CM^TXL^, indicating that the increased phosphorylation of p70s6k1 was required for activation of the eNOS pathway under our conditions. Because of the above-mentioned effects induced by p70s6k1 siRNA, no difference in the percentage of dead CMECs was observed between the CM^TXL^+siRNA and Control groups (37.20 ± 0.85% in CM^TXL^ + siRNA group vs 37.47 ± 1.54% in the Control group, P > 0.05) **(Figure 5F-G)**. The siRNA against p70s6k1 alone did not increase the cell death of CMECs during H/R (36.27 ± 1.17% vs 37.47 ± 1.54% in the Control group, P > 0.05), as shown in **Figure S17**. The above findings were further confirmed with a second siRNA construct against p70s6k1 **(Figure S18)**. Therefore, we concluded that CM^TXL^ reduced the H/R-induced injury of CMECs by promoting the expression of the p70s6k1, and a subsequent increase in the level of p-p70s6k1, thus activating eNOS pathway.

### CM-derived sEVs mediate eNOS activation by increasing p70s6k1 in CMECs through reduction of miR-145-5p

**Figure 6:**
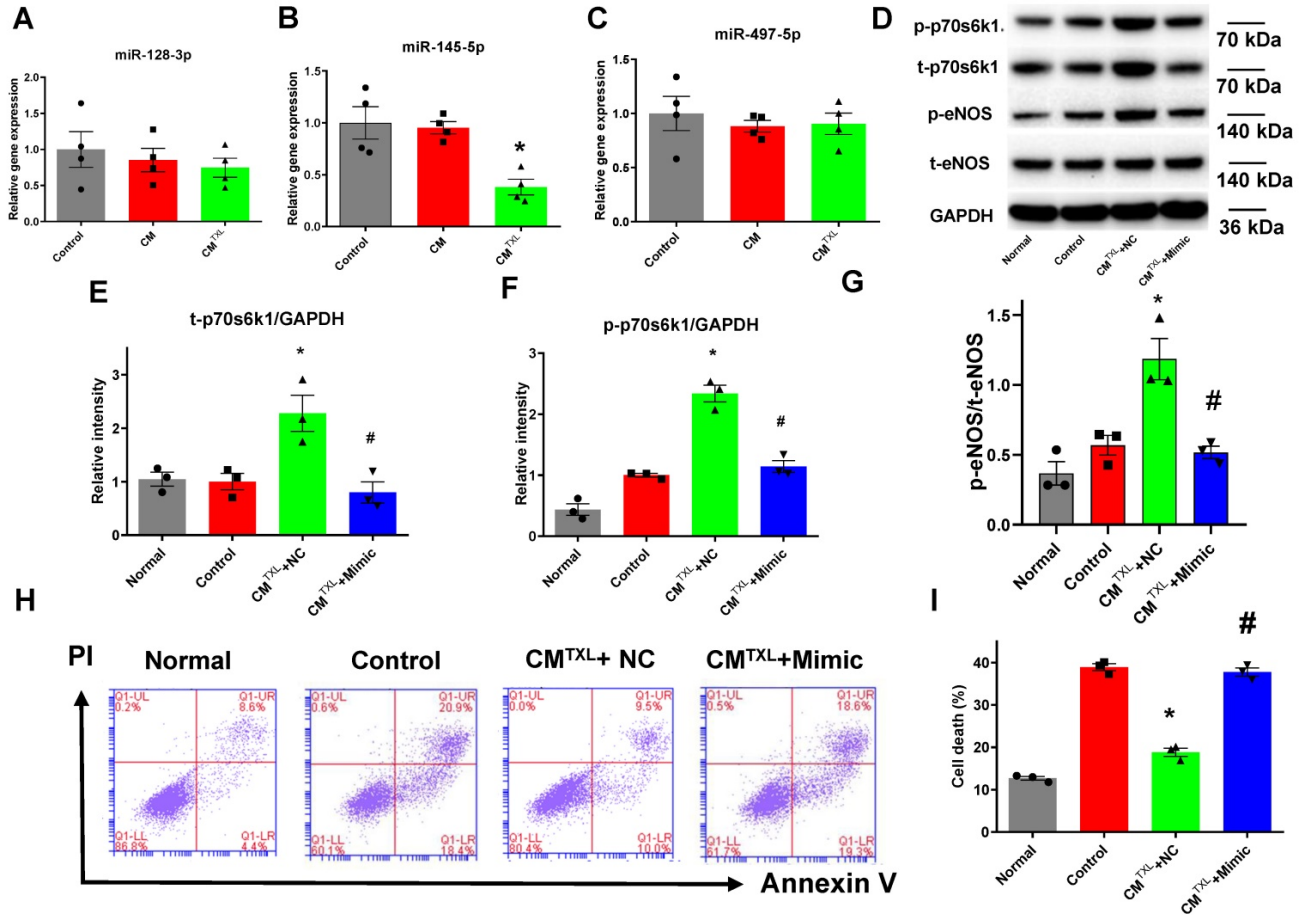
CM-derived sEVsmediate eNOSactivation by increasing p70s6k1 in CMECs through reduction of miR-145-5p. A-C) The level of microRNAs targeting the p70s6k1 mRNA in CMECs (n = 4). D-G) The levels of p-70s6k1, t-p70s6k1, p-eNOSand t-eNOSwere detected by Western Blot (n=3). H-I) Dead CMECs in flow cytometry assay (n=3). * P<0.05 vs. Control; # P <0.05 vs. CM^TXL^+NC. sEVs: small extracellular vesicles; CM: cardiomyocyte; CM^TXL^: TXL-pretreated CMs; CM^TXL^+Mimic group: CMECs were transfected with miR-145-5p mimics before being co-cultured with CM^TXL^ and then exposed to H/R; CM^TXL^+NC: CMECs were transfected with negative control of mimics before being co-cultured with CM^TXL^ and then exposed to H/R. All data are mean ±SEM. Statistical analysis was performed with one-way ANOVA followed by Tukey's test.

Our previous research demonstrated that treating CMs with TXL promoted the expression of p70s6k1 via the inhibition of miR-128-3p 18. Therefore, we measured the level of miR-128-3p in CMECs co-cultured with or without CM^TXL^ in the co-culture system after being exposed to H/R. Surprisingly, no change in the miR-128-3p level was observed in CMECs co-cultured with CM^TXL^ after H/R, compared with CMECs co-cultured with or without CM (0.75 ± 0.13% vs 1.00 ± 0.25% in the Control group and 0.85 ± 0.16% in the CM group, P > 0.05) **(Figure 6A)**. Next, we measured the levels of two other microRNAs, miR-145-5p 39 and miR-497-5p 40, which were reported to target the mRNA of p70s6k1. No change was observed in miR-497-5p levels under any of the conditions **(Figure 6C)**. However, CMECs co-cultured with CM^TXL^ had a lower level of miR-145-5p as compared with cells co-cultured with or without CM (0.38 ± 0.07% vs 1.00 ± 0.16% in the Control group and 0.95 ± 0.06% in the CM group, P < 0.05) **(Figure 6B),** confirmed by the *in vivo* results that the level of miR-145-5p was lower in the rat adult CMEC isolated from the TXL-pretreated hearts (0.32 ± 0.04% vs 1.00 ± 0.11% in the Control group, P < 0.05) **(Figure S16F)** after myocardial I/R. To investigate whether the downregulation of miR-145-5p was necessary in the beneficial effects of TXL against H/R-induced cell death, miR-145-5p mimics were utilized. CMECs were transfected with miR-145-5p mimics or control, and then co-cultured with CM^TXL^ when subjected to H/R. Western blotting analysis demonstrated that miR-145-5p mimics downregulated the expression of p70s6k1 (0.80 ± 0.20 in CM^TXL^ + Mimic group vs 2.28 ± 0.19 in the CM^TXL^ +NC group, P < 0.05) **(Figure 6D-E)** and thus decreased its phosphorylation (1.14 ± 0.10 in CM^TXL^ + Mimic group vs 2.34 ± 0.14 in the CM^TXL^ +NC group, P < 0.05) **(Figure 6D, F)**, with a significant reduced eNOS activity (0.52 ± 0.04 in CM^TXL^ + Mimic group vs 1.19 ± 0.15 in the CM^TXL^ +NC group, P < 0.05)** (Figure 6D, G)**. As a consequence of the decrease in the p-eNOS level in CMECs during H/R, being co-cultured with CM^TXL^ did not reduce cell death in CMECs (37.77 ± 0.98% in CM^TXL^ + Mimic group vs 38.90 ± 0.83% in the Control group, P > 0.05)** (Figure 6H-I)**, indicating that the upregulation of miR-145-5p abolished the beneficial effects of CM^TXL^ on CMECs. As shown in **Figure S19**, transfection of miR-145-5p mimics into CMECs had negligible effects on the cell death of CMECs after H/R (37.57 ± 1.15% vs 35.33 ± 1.00% in the Control group, P > 0.05). These results suggested that the decrease of miR-145-5p in CMECs mediated the protective effect of CM^TXL^ on CMECs during H/R.

### SEV transfer of linc-ROR from CMs activates the eNOS pathway by promoting the expression of p70s6k1 in CMECs

**Figure 7:**
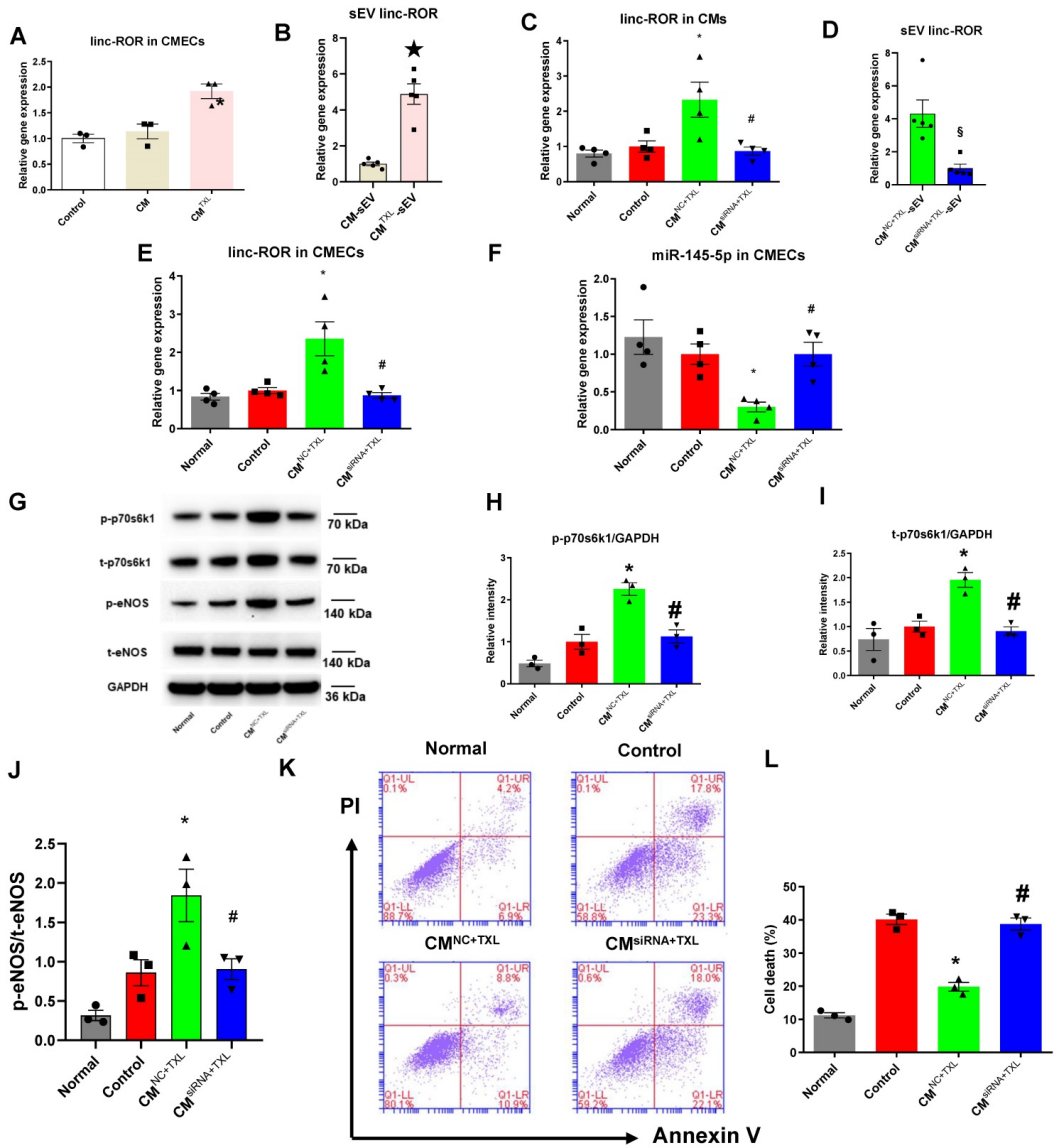
SEV transfer of linc-ROR from CMs activates the eNOSpathway by promoting the expression of p70s6k1 in CMECs. A) The level of linc-ROR in CMECs co-cultured with or without CM (or CM^TXL^) (n=3). B) The level of linc-ROR in sEVsderived from TXL-pretreated and non-TXL-pretreated CMs after H/R (n=5). C-K) CMs were transfected with siRNA against linc-ROR or siRNA negative control as indicated, and then pretreated with TXL for 1 h before being co-cultured with CMECs in the co-culture system under normal or H/R condition. RNA and proteins were isolated for RT-PCR or WB. C) The level of linc-ROR in CMs (n=4). D) The level of linc-ROR in sEVsfrom CMs (n=5). E) The level of linc-ROR in CMECs (n=4). F) The level of miR-145-5p in CMECs (n=4). G-J) The levels of p-70s6k1, t-p70s6k1, p-eNOSand t-eNOSwere detected by Western Blot (n=3). K-L) Dead CMECs in flow cytometry assay (n=3). * P<0.05 vs. Control; ☆P <0.05 vs. CM-sEV; # P <0.05 vs. CM^NC+TXL^; §P<0.05 vs. CM^NC+TXL^-sEV. sEV: small extracellular vesicles; CM: cardiomyocyte; CM^NC+TXL^: CMs having been transfected with negative control of siRNA and then pretreated with TXL; CM^siRNA+TXL^: CMs having been transfected with linc-ROR siRNA and then pretreated with TXL; CM^NC+TXL^ group: CMECs were co-cultured with CMNC+TXL and subjected to H/R; CM^siRNA+TXL^ group: CMECs were co-cultured with CM^siRNA+TXL^ and subjected to H/R. All data are mean ±SEM. Statistical analysis was performed with one-way ANOVA followed by Tukey's test for multiple comparison or unpaired Student T-test for two-group comparison

SEVs transfer cargos such as proteins and RNAs (including mRNAs, lncRNAs and microRNAs). Therefore, we hypothesized that the sEVs released from CM^TXL^ shuttled a protective substance (or substances) from CM^TXL^ to CMECs during H/R. Competing endogenous RNAs (ceRNAs) regulate other RNA transcripts by functioning as a kind of “sponge” for microRNAs, sequestering these microRNAs from their normal targets 41, 42. lncRNAs are important members of the ceRNA family and have been frequently reported to regulate gene expression by sequestering microRNAs 41. A recent study reported that sEV transfer of one such lncRNA, linc-ROR, promoted cell survival among hepatocellular cancer cells during hypoxia by targeting and downregulating miR-145-5p 43. Therefore, we measured the level of linc-ROR in CMECs co-cultured with CM^TXL^ under H/R conditions and found a significant augmentation of linc-ROR compared with those cultured with or without CMs (1.92 ± 0.14% vs 1.00 ± 0.08% in the Control group and 1.14 ± 0.14% in the CM group, P < 0.05) **(Figure 7A)**. Furthermore, CM^TXL^-sEVs contained more linc-ROR than CM-sEVs (4.88 ± 0.57% in the CM^TXL^-sEV group vs 1.00 ± 0.10% in the CM-sEV group, P < 0.05) **(Figure 7B)**. Similarly, we found that TXL pretreatment could increase the level of linc-ROR (1.96 ± 0.15% vs 1.00 ± 0.11% in the Control group, P < 0.05) **(Figure S16G)** in the adult rat CMECs isolated from the hearts having been stressed by I/R, and GW4869 treatment could reverse this trend (0.83 ± 0.06% vs 1.00 ± 0.11% in the Control group, P > 0.05) **(Figure S16G)**. To determine whether linc-ROR mediates the protective effect of CM^TXL^-sEVs on CMECs during H/R, linc-ROR siRNA was used to silence linc-ROR in CMs before being pretreated with TXL (CM^siRNA+TXL^) and exposed to H/R. As shown in **Figure 7C**, transfection of linc-ROR siRNA into CMs significantly decreased the level of linc-ROR in CMs (0.87 ± 0.11% in the CM^siRNA+TXL^ group vs 2.33 ± 0.50% in the CM^NC+TXL^ group, P < 0.05), leading to a reduction of the level of linc-ROR in sEVs derived from CM^TXL^(1.00 ± 0.25% in the CM^siRNA+TXL^-sEV group vs 4.31 ± 0.83% in the CM^ NC+TXL^-sEV group, P < 0.05) **(Figure 7D)**. The reduced level of sEV linc-ROR from CM^TXL^ led to the unchanged levels of linc-ROR in CMECs when co-cultured with CM^TXL^ (0.87 ± 0.07% in the CM^siRNA+TXL^ group vs 1.00 ± 0.08% in the Control group, P > 0.05) **(Figure 7E)**. As a result, the level of miR-145-5p in CMECs co-cultured with CM^siRNA+TXL^ were similar to that in CMECs of the Control group (1.00 ± 0.16% in the CM^siRNA+TXL^ group vs 1.00 ± 0.14% in the Control group, P > 0.05) **(Figure 7F)**. Correspondingly, the increase of p-p70s6k1 (1.13 ± 0.16 in the CM^siRNA+TXL^ group vs 1.00 ± 0.17 in the Control group, P > 0.05) **(Figure 7G-H)**, t-p70s6k1 (0.91 ± 0.09 in the CM^siRNA+TXL^ group vs 1.00 ± 0.11 in the Control group, P > 0.05) **(Figure 7G, I)** and the ratio of p-eNOS/t-eNOS (0.91 ± 0.13 in the CM^siRNA+TXL^ group vs 0.86 ± 0.17 in the Control group, P > 0.05) **(Figure 7G, J)** in CMECs co-cultured with CM^TXL^ was prevented once CMs were transfected with linc-ROR siRNA before being pretreated with TXL. As a consequence of all these changes induced by the transfection of linc-ROR siRNA into CMs, the percentage of dead CMECs in the CM^siRNA+TXL^ group was similar to that in the Control group (38.73 ± 1.78% in the CM^siRNA+TXL^ group vs 40.13 ± 1.55% in the Control group, P > 0.05) **(Figure 7K-L)**. Furthermore, coculturing CMECs with CMs transfected with siRNA against linc-ROR did not affect the death percentage of CMECs under H/R conditions (37.83 ± 1.58% vs 40.13 ± 1.55% in the Control group, P > 0.05)** (Figure S20A-B)**. These data demonstrated that CM^TXL^ transferred linc-ROR to CMECs via sEVs, which protected CMECs during H/R.

## Discussion

The crosstalk between CMECs and CMs has emerged as a key player in heart development and during the onset and progression of heart diseases 6. Anatomically, CMECs closely surround CMs to form “perfusion units”, with a small distance between these two types of cells (approximately 1-2 μm) 5, 44. The close proximity between CMs and CMECs allow them to communicate with each other in a paracrine manner, or in some certain scenarios, even through direct cell-to-cell contact 6. Despite an increasing number of studies showing that endothelial cells can protect CMs during I/R 7, 8, 9, whether CMs are capable of sending messengers to CMECs and thus blunting myocardial I/R injury remains unknown. Here, we showed that cardiomyocyte-derived sEVs can enhance eNOS activity in CMECs to protect against I/R injury. Our study demonstrated that CMEC derived eNOS has an indispensable role in the protection induced by TXL against I/R injury. This beneficial effect is mediated in part by the transfer of sEV linc-ROR from CM^TXL^ to CMECs. The transfer of linc-ROR to CMECs decreased miR-145-5p levels. Downregulation of miR-145-5p increased the expression of p70s6k1 and augmented its phosphorylation, thereby stimulating the eNOS pathway and promoting NO production, improving microcirculation and attenuating I/R injury. To the best of our knowledge, this is the first study demonstrating that CMs, stimulated by agents such as TXL, protect CMECs via sEVs during H/R (I/R).

In hearts, CMECs outnumber CMs by 3:1, despite the mass ratio of CMEC-to-CM being approximately 0.04 45. CMECs have a key role in the microcirculation by providing nutrition and oxygen, and they also release protective substances to impact CMs under certain conditions. It was reported that the dysfunction/death of CMECs leads to no-reflow phenomena after reperfusion 46, and that neuregulin secreted by CMECs protected CMs from I/R injury 7, demonstrating the important role of CMECs in I/R injury. Furthermore, CMECs are more vulnerable to I/R, during which the apoptosis of CMECs precedes that of CMs 47. Therefore, protecting CMECs during I/R might be a method to reduce I/R injury. Indeed, our data suggest a reciprocal benefit whereby CM-derived sEVs can activate eNOS in CMECs, thus protecting CMECs, as well as CMs, from I/R injury.

Tongxinluo (TXL) is a traditional Chinese medicine that was registered with the China State Food and Drug Administration (CFDA) in 1996 18. The major ingredients of TXL are extracted from Radix ginseng, Buthus martensi, Hirudo, Eupolyphaga seusteleophaga, Scolopendra subspinipes, Periostracum cicadae, Radix paeoniae rubra, Semen ziziphispinosae, Lignum dalbergiae odoriferae, Lignum santali albi, and Borneolum syntheticum 48. Its active constituents include peoniflorin, ginsenoside Rg1, ginsenoside Rb1, jujuboside A, jujuboside B, isoborneol and borneol 49. Although our previous studies suggested that TXL exerted its cardioprotective effects by activating the eNOS pathway 16, 17, it remained unclear whether CMEC derived eNOS was involved in the TXL-induced cardioprotection. In the present study, disrupting the function of eNOS in CMECs with Triton X-100 largely offset the cardioprotective effect of TXL, indicating that CMEC eNOS is required by TXL to exert its cardioprotective effects. As Triton X-100 is a detergent, the risk exists that it may completely destroy endothelial cell function and microvascular vessel structure, stopping TXL from going through microvascular vessels to impact cardiomyocytes. However, in our study, we found that Triton-X 100 at a concentration of 0.125 % was able to reduce NO production, without affecting the increased phosphorylation of eNOS induced by TXL, suggesting cellular processes were still occurring. The fact that TXL could still facilitate eNOS phosphorylation in the *ex vivo* hearts treated with Triton-X 100 indicates that Triton-X 100 could cross the microvascular vessels and impact cardiomyocytes, as TXL alone could not directly activate eNOS in CMECs. If cardiac endothelium had been completely destroyed by Triton-X 100 and had not allowed TXL to go through it and reach cardiomyocytes, the p-eNOS could not have been increased in the whole hearts. This was consistent with the results from other groups that low levels of Triton-X 100 pretreatment was effective in abolishing coronary endothelial eNOS-mediated NO production with negligible effects on myocardial function and infarct size 9, 23, 50. The indispensable role of CMEC eNOS in cardioprotection has also been suggested before. For example, ECs rather than CMs had a key role in eNOS-dependent cardioprotection induced by exercise training 9. Additionally, our results are consistent with another study reporting that eNOS in ECs plays a critical role in ischemic preconditioning-induced cardioprotection 23.

ENOS is a membrane protein mainly found in plasma membrane caveolae or on the Golgi apparatus 51. The activity of eNOS can be regulated by a variety of posttranslational modifications, such as acylation, nitrosylation and phosphorylation. Among these, phosphorylation is the major way that influences eNOS enzyme activity 51. The biological effects of eNOS activation also depend on whether eNOS is coupled or not 52. When the dihydrobiopetrin (BH2) to tetrahydrobiopetrin (BH4) ratio is increased, eNOS is not coupled to L-arginine oxidation and therefore produces superoxide (SO). SO is subsequently converted to hydrogen peroxide, leading to a burst production of ROS 53. Whereas the coupled eNOS generates NO in the presence of BH4 by promoting the reduction of oxygen to L-arginine oxidation. It is worth noting that the increase of NO in cells or tissues does not necessarily mean beneficial effects in pathological conditions such as ischemia or I/R 53. High concentrations of NO can cause cell death 54, 55 and depress contractile function 56, while small concentrations of NO, which can be induced by certain treatments such as antioxidants 57, improve ventricular function 58 and confer protection on the hearts during I/R 59. Here, we found that TXL could alleviate I/R injury by activating eNOS and thus increasing the level of NO, whereas this TXL-induced protective effects were totally offset by L-NNA, an eNOS inhibitor that inhibited eNOS activity and reduced NO generation. These data suggest that, the augmented eNOS phosphorylation and NO level, which were induced by TXL, could protect the hearts from I/R injury.

In our study, we found a novel pathway of crosstalk between CMs and CMECs during I/R, whereby the indirect activation of eNOS in CMECs is enough to induce cardioprotection. Previously, we have shown that TXL activated eNOS in the heart 16, 17. However, in the present study our *in vitro* experiments showed that TXL had a mild but significant protection against H/R-induced injury of CMECs without significantly facilitating the phosphorylation of eNOS, and that an inhibitor of eNOS, L-NNA, failed to abrogate this beneficial effect of TXL. This suggested that TXL treatment on its own does not directly activate eNOS in CMECs. In contrast, the CMEC-CM co-culture model demonstrated that CMECs cultured with CM^TXL^ had a strong protection against H/R injury, dependent of the enhanced eNOS activity. Importantly, the effects were reversed if CM were treated with GW4869, which blocks the generation of sEVs. The role of sEVs in mediating the beneficial effect of TXL was further validated by the result that CMECs treated with CM^TXL^-sEVs had significantly higher levels of p-eNOS and fewer dead cells. Finally, in endothelium-permeabilized hearts, where brief low levels of Triton X-100 were used to abolish eNOS-mediated NO generation in endothelial cells, the infarct-limiting effect of TXL was largely offset. These data support a model where crosstalk between the preconditioned-CMs and CMECs result in reciprocal protection against I/R injury.

SEVs are double-layer extracellular vesicles between 30-100 nm in size 35. They can be secreted by most cells and have been found in most body fluids, including saliva, urine and plasma 60. These nanosized particles are considered to transfer proteins, microRNA and long non-coding RNA cargos through the bloodstream and other body fluids, protecting them from enzymatic degradation 35***.***The amounts and contents of sEVs vary depending on the type of parent cell stimuli (e.g. alcohol 61, hypoxia 62, hypoxia/reoxygenation 61). Regarding intercellular communication, sEVs impact target cells in different ways, such as directly transferring their biochemical contents to target cells after being taken up 35. In the “perfusion units”, CMECs and CMs are adjacent to each other anatomically in a heart, therefore sEVs released from these two types of cells can significantly influence one another because of the relatively high local concentration of sEVs 5, 44. Indeed, many studies have demonstrated that sEVs can mediate crosstalk between CMECs and CMs under certain conditions 36, 63. Of note, sEVs released from endothelial cells treated with ischemic preconditioning protected cardiomyocytes under H/R conditions 8. In our study, using GW4869, we found it was CM^TXL^-sEVs that exerted the protective effects of CM^TXL^ on CMECs during H/R. Although GW4869 is an inhibitor of neutral sphingomyelinase as well as sEV biogenesis, GW4869 on its own had no effect on cell death of CMECs and CMs under our conditions, suggesting against an off-target effect induced by inhibiting the neutral sphingomyelinase. In isolation this experiment alone would not be enough to show that sEVs are involved in the phenotype. Taking this into consideration, we isolated sEVs from CM^TXL^ and demonstrated that these vesicles were capable of protecting endothelial cells from H/R injury. Furthermore, we found that sEV-depleted conditioned medium from CM^TXL^ didn't affect the cell death of CMECs during H/R. Another point that needs attention is that the cardioprotective effect of TXL didn't entirely depend on the CM^TXL^-sEVs, as *in vivo* administration of GW4869 largely attenuated, but did not completely block the infarct-sparing effect of TXL. This may be due to the direct protection of TXL on CMECs 25 and CMs 18 during H/R (I/R). In summary, our findings may contribute to deeper insights into the crosstalk between CMs and CMECs during I/R.

P70s6k is a member of the AGC family and is regulated by the mammalian target of rapamycin (mTOR) pathway 64. It was demonstrated that p70s6k plays important roles in various biological processes, such as protein synthesis, glucose homeostasis, and cell survival 65. Previous studies reported that the p70s6k protein is involved in the cardioprotective RISK pathway and might be phosphorylated by the RISK-related proteins Erk and Akt 66. There are two subtypes of p70S6K, p70S6K1 and p70S6K2 67, with the latter being detected at very low levels in adult hearts 68. eNOS has a cardioprotective role during I/R 9, 23 and although facilitating p70s6k phosphorylation with cysteine-rich, angiogenic inducer 61 or neuropeptide Y activated the eNOS pathway 37, 38, it remains unclear whether activating the p70s6k1/eNOS pathway during I/R is beneficial. Our current and previous studies demonstrated that activation of the eNOS pathway was indispensable for TXL-induced cardioprotective effects 16, 17. In this study, we found that CMECs co-cultured with CM^TXL^ had significantly higher levels of steady-state p70s6k1 and consequently, its phosphorylated form. Furthermore, knocking down p70s6k1 prevented the increased phosphorylation of eNOS induced by TXL in CMECs during H/R, with the inhibitory effects on cell death being abrogated. This indicated that p70s6k1 is required for the phosphorylation of eNOS in cardioprotection induced by certain agents such as TXL. To the best of our knowledge, this is the first study to unravel the role of the p70s6k1/eNOS pathway in I/R injury.

MicroRNAs, a family of small non-coding single-stranded RNAs, are emerging as robust molecules that modulate the post-transcriptional expression of genes 69. In our CMEC-CM co-culture model, the upregulation of steady-state levels of p70s6k1 in CMECs co-cultured with CM^TXL^ was correlated with the reduction of miR-145-5p, which targets and represses the translation of p70s6k1 mRNA 43. Our further experiments showed that transfecting CMECs with miR-145-5p mimics prevented the upregulation of p70s6k1 in CMECs co-cultured with CM^TXL^. Following the inhibition of the p70s6k1/eNOS pathway, CM^TXL^ no longer protected CMECs from H/R-induced injury in the co-culture system. Therefore, the downregulation of miR-145-5p mediated the beneficial effects of CM^TXL^ on CMECs. This finding is partly consistent with a recent study where suppression of miR-145-5p attenuated acute cerebral ischemia/reperfusion injury in rats 70. Two other studies also showed that the overexpression (inhibition) of miR-145-5p promoted (repressed) the apoptosis of hepatocellular carcinoma cells *in vitro* 43, 71. Previously, we showed that treating CMs with TXL directly could reduce the level of miR-128-3p in CMs after H/R 18. However, in the current study, we found that the expression of miR-145-5p in CMECs, but not miR-128-3p, was decreased after CMECs took up CM^TXL^-sEVs (rich in linc-ROR) under the condition of H/R. As the effects of these two studies resulted from two different treatments (linc-ROR-rich CM^TXL^-sEVs or TXL alone), it is understandable that the trends in the change of miR-128-3p were not the same in these two studies.

lncRNAs are a type of RNA, defined as transcripts with lengths exceeding 200 nucleotides that are not translated into proteins 72. lncRNAs have important roles in remodeling chromatin and genome architecture, RNA stabilization and transcription regulation, including enhancer-associated activity 73. lncRNA can function as ceRNAs that sequester specific miRNAs from their mRNA targets, thereby increasing the expression of target mRNAs 73. In the current study, the downregulation of miR-145-5p in CMECs co-cultured with CM^TXL^ was attributable to the increased level of linc-ROR, a lncRNA transferred from CM^TXL^ via sEVs. This can explain the phenomenon that the beneficial effects of CM^TXL^-sEVs disappeared after excessive miR-145-5p mimics were transfected into CMECs to overwhelm the binding capacity of linc-ROR. Our finding is in line with a prior study, where the sEV transfer of linc-ROR promoted the survival of liver cancer cells during hypoxic stress via the downregulation of miR-145-5p 43. Furthermore, linc-ROR was recently been reported to alleviate hypoxia-induced damage by downregulating miR-145-5p in H9c2 cells 74. Another study reported that linc-ROR attenuated cobalt chloride-induced hypoxia injury by inhibiting miR-145-5p 75.

Our study uncovers a new pathway of crosstalk in regulating cardioprotection during I/R injury. It adds to a growing literature that demonstrates the co-dependency of the “perfusion unit” cells in the heart in response to injury and stress. The major limitation of our study is that, further work will need to be done to understand the mechanism responsible for the increase of sEV linc-ROR from CM^TXL^ during I/R and whether this occurs through the transcription of this gene in CMs or, enhanced packaging of linc-ROR from CMs to sEVs. Another limitation is that mice with cardiomyocyte-specific knockout of linc-ROR (or endothelium-specific knockout of miR-145-5p) were not used in our study to validate the therapeutic values of our study. However, it is clear from this study and others that the emerging role for crosstalk between CMs and CMECs, in physiological adaptations as well as pathological responses, has strong potential for therapeutic targeting.

## Supplementary Material

Supplementary figures.

## Abbreviations

AAR: area-at-risk
AMI: acute myocardial infarction
CeRNAs: competing endogenous RNAs
CMECs: cardiac microvascular endothelial cells
CMs: cardiomyocytes
eNOS: endothelial nitric oxide synthase
H/R: hypoxia/reoxygenation
IA: infarcted area
I/R: ischemia/reperfusion
LAD: left anterior descending artery
Linc-ROR: long intergenic non-protein coding RNA, regulator of reprogramming
PBS: phosphate buffered saline
PI: propidium iodide
siRNA: small interfering RNA
TEM: transmission electron microscopy
TTC: 2, 3, 5-triphenyltetrazolium chloride
TXL: tongxinluo
