# Supplementary material for: Cardiomyocyte-derived small extracellular vesicles can signal eNOS activation in cardiac microvascular endothelial cells to protect against Ischemia/Reperfusion injury
Source: Theranostics. 2020 Sep 23;10(25):11754–74. doi: 10.7150/thno.43163 (PMC7546010; doi:10.7150/thno.43163)
Supplement: Supplementary file 1 — Supplementary figures. [file thnov10p11754s1.pdf]

Figure S1

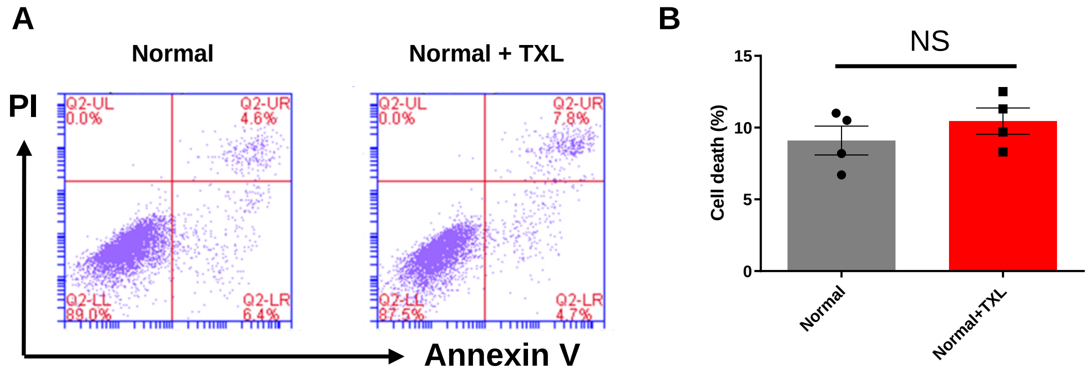

TXL at a concentration of 800 μg/mL did not affect the viability of CMECs under normal condition. A) Representative scatter diagram of the death of CMECs treated with TXL or not in flow cytometry assay (n=4). B) Quantitative analysis for dead cells in Flow Cytometry Assay. TXL: Tongxinluo; CMECs: Cardiac microvascular endothelial cells. All data are mean ± SEM. Statistical analysis was performed with unpaired Student T-test.

Figure S2

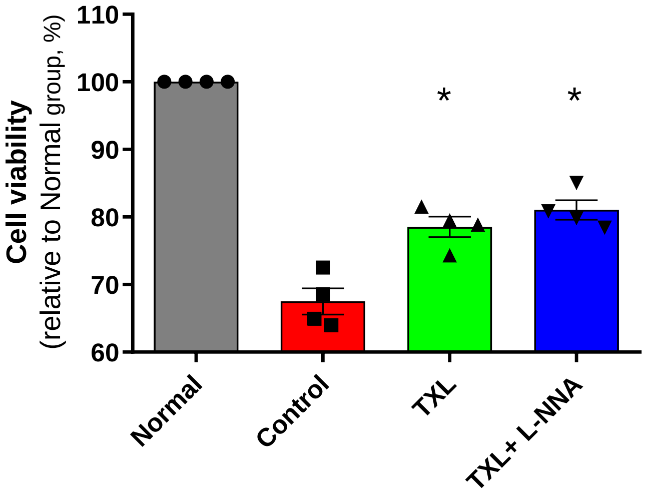

L-NNA can not inhibit the direct protective effects of TXL on CMECs during H/R in the CCK-8 assay (n=4). *P<0.05 vs. Control; CMECs: Cardiac microvascular endothelial cells; H/R: Hypoxia/Reoxygenation; CCK-8: Cell counting kit-8. All data are mean ± SEM. Statistical analysis was performed with one-way ANOVA followed by Tukey’s test.

Figure S3

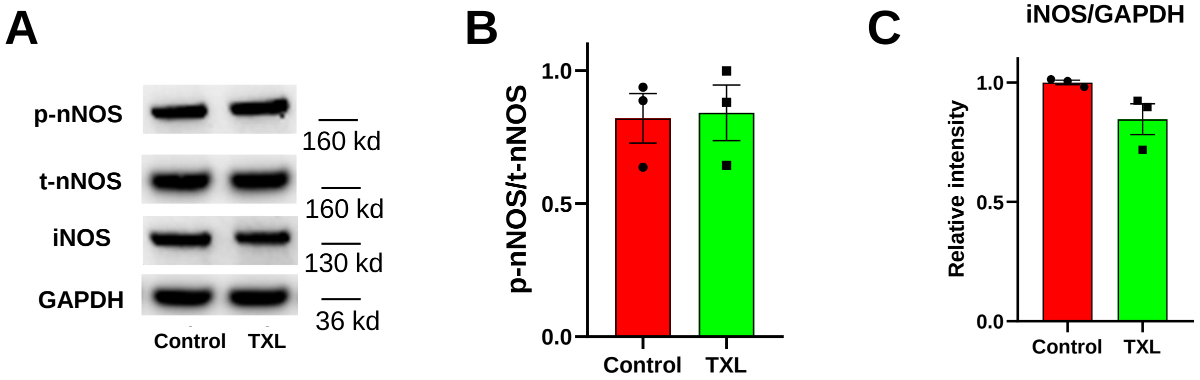

The effects of TXL on the activity of nNOS and iNOS in the ex-vivo hearts under I/R. A-C) The levels of p-nNOS, t-nNOS and iNOS were detected by Western Blot (n=3). TXL: Tongxinluo;. All data are mean ± SEM. Statistical analysis was performed with unpaired Student T-test.

Figure S4

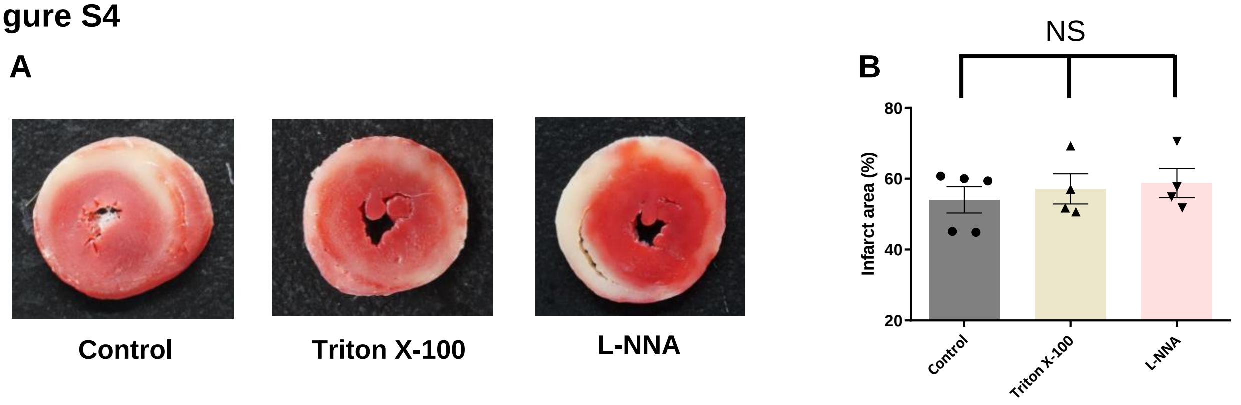

Triton X-100 or L-NNA alone did not increase infarct size after I/R in isolated rat hearts. A) Representative images of TTC staining in each group (n = 4-5). The red-stained areas indicate viable tissue, and the pale white areas indicate infarct tissue. B) Quantitative analysis for infarct size in each group. I/R: Ischemia/Reperfusion. All data are mean ± SEM. Statistical analysis was performed with one-way ANOVA followed by Tukey’s test.

Figure S5

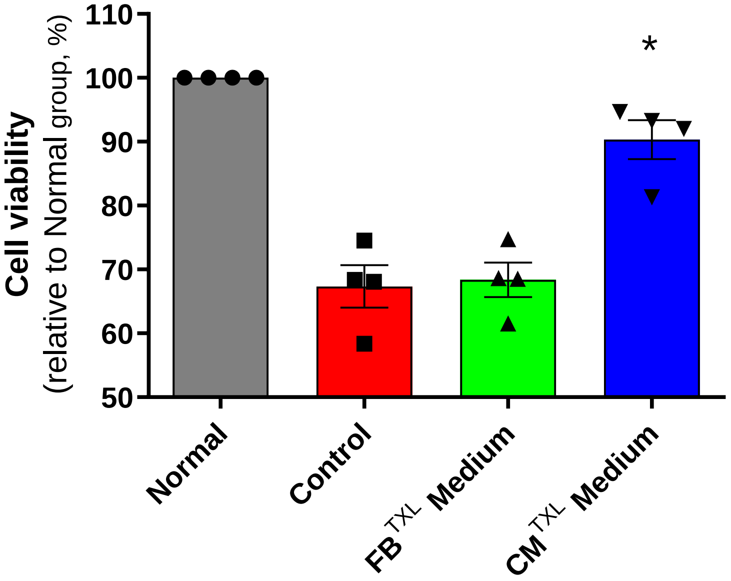

CM^{TXL} medium could increase the viability of CMECs during H/R in the CCK-8 assay (n=4). *P<0.05 vs. Control; CM: cardiomyocyte; CM^{TXL}: TXL-pretreated cardiomyocyte; CMECs: Cardiac microvascular endothelial cells; H/R: Hypoxia/Reoxygenation; CCK-8: Cell counting kit-8. All data are mean ± SEM. Statistical analysis was performed with one-way ANOVA followed by Tukey's test.

Figure S6

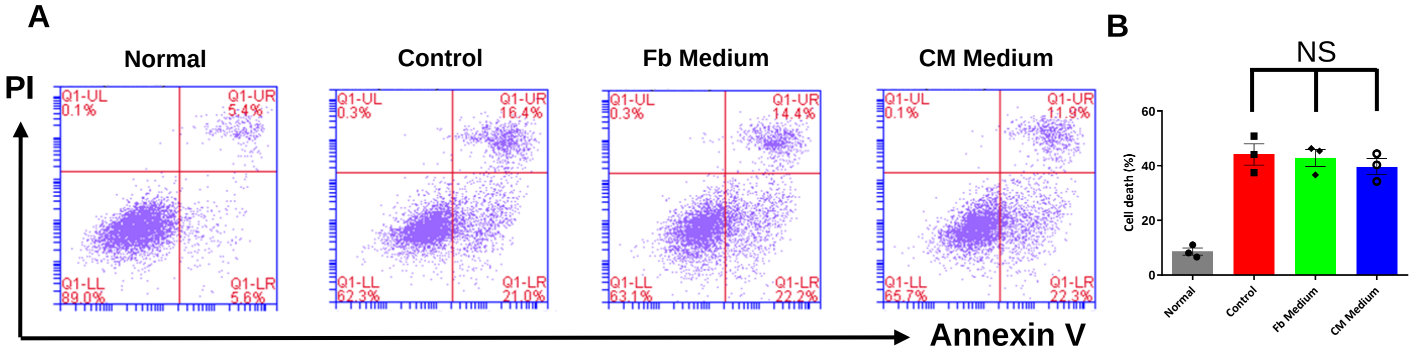

Conditioned medium from Fb or CM without being pretreated with TXL didn't affect the death of CMECs during H/R. A) Representative scatter diagram of the death of CMECs with different treatments in flow cytometry assay (n=3). B) Quantitative analysis for dead cells in Flow Cytometry Assay. CMECs: Cardiac microvascular endothelial cells; Fb Medium: conditioned medium collected from fibroblasts having been subjected to Hypoxia 18h/Reoxygenation 2h; CM medium: conditioned medium collected from cardiomyocytes having been subjected to Hypoxia 18h/Reoxygenation 2h. All data are mean ± SEM. Statistical analysis was performed with one-way ANOVA followed by Tukey's test.

Figure S7

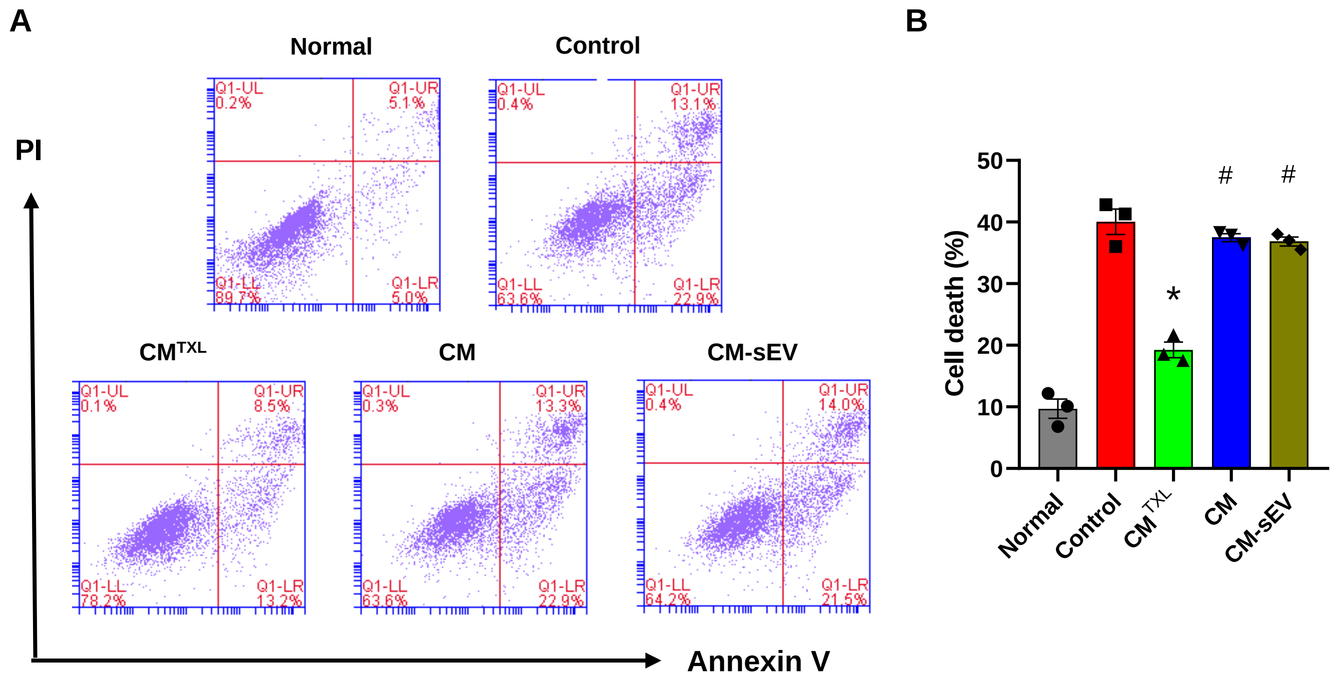

CM or CM-sEV did not affect the death of CMECs under H/R. A-B) Dead CMECs in flow cytometry assay (n=3). *$P<0.05$ vs. Control; #$P<0.05$ vs. CM^{TXL}. CM: cardiomyocyte; CM^{TXL}: TXL-pretreated cardiomyocyte; sEV: small extracellular vesicles; CM-sEV: sEVs derived from CM. All data are mean ± SEM. Statistical analysis was performed with one-way ANOVA followed by Tukey's test.

Figure S8

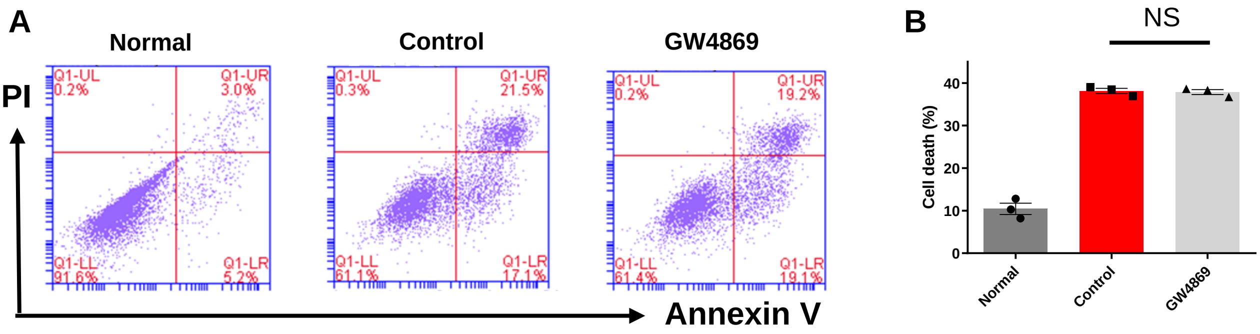

GW4869 alone did not promote the death of CMECs after H/R. A) Representative scatter diagram of the death of CMECs after being treated with GW4869 or not in flow cytometry assay (n=3). B) Quantitative analysis for dead cells in Flow Cytometry Assay. CMECs: Cardiac microvascular endothelial cells; H/R: Hypoxia/Reoxygenation. All data are mean ± SEM. Statistical analysis was performed with one-way ANOVA followed by Tukey’s test.

Figure S9

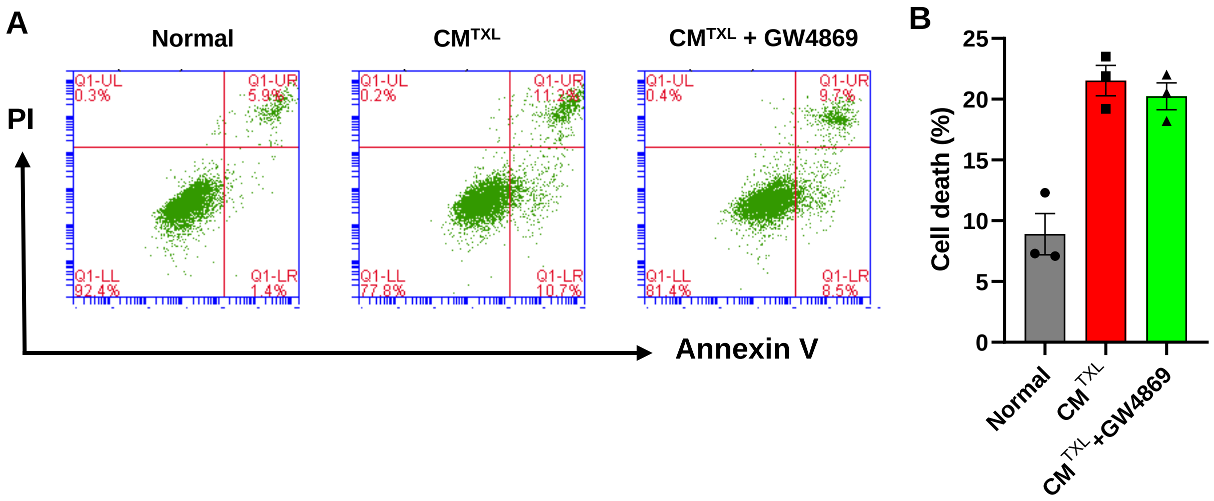

GW4869 alone did not increase the death of CM^{TXL} after H/R. A) Representative scatter diagram of cell death in CM^{TXL} treated with GW4869 or not in flow cytometry assay (n=3). B) Quantitative analysis for dead CM^{TXL} in Flow Cytometry Assay. CM^{TXL}: CM pretreated with TXL; H/R: Hypoxia/Reoxygenation. All data are mean ± SEM. Statistical analysis was performed with one-way ANOVA followed by Tukey's test.

Figure S10

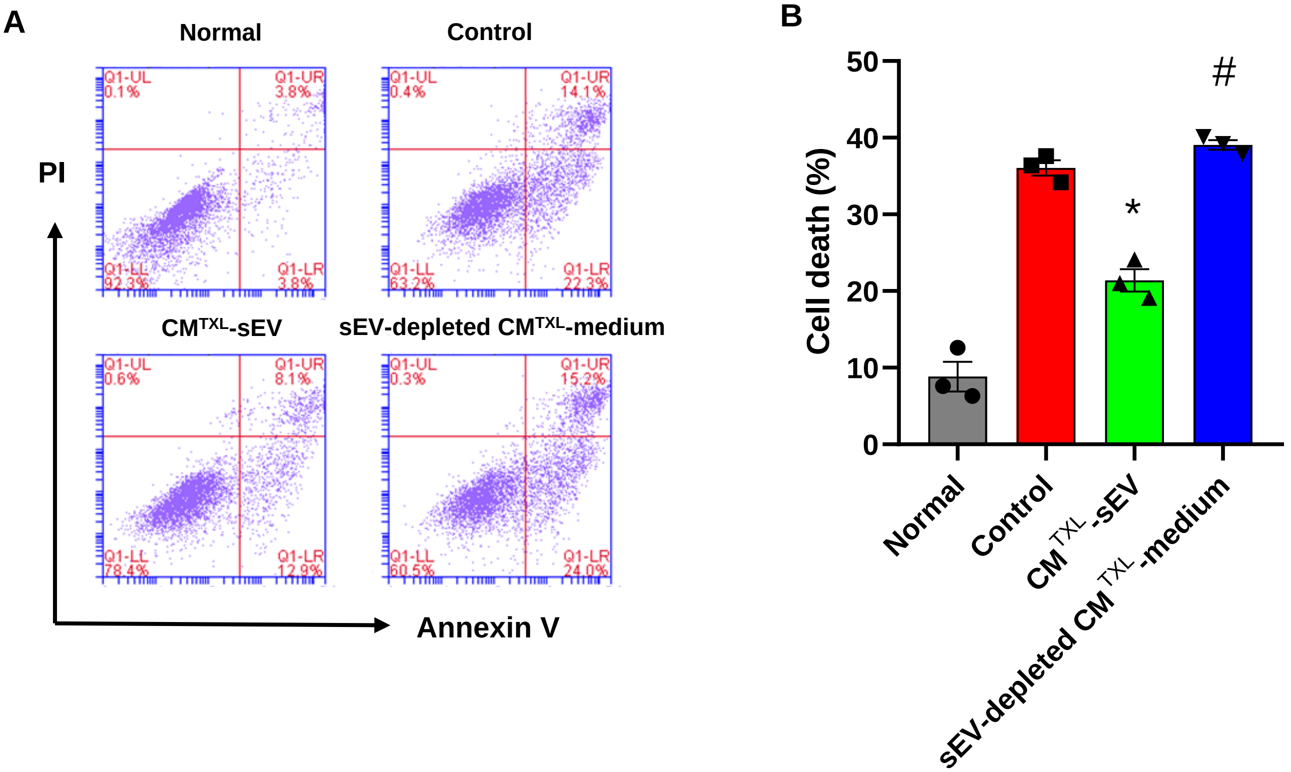

SEV-depleted conditioned medium from CM^{TXL} didn't affect the cell death of CMECs during H/R. A) Representative scatter diagram of the death of CMECs with different treatments in flow cytometry assay (n=3). B) Quantitative analysis for dead cells in Flow Cytometry Assay. *P<0.05 vs. Control; #P <0.05 vs. CM^{TXL}-sEV. sEV: small extracellular vesicle; CMECs: Cardiac microvascular endothelial cells; CM: cardiomyocyte; CM^{TXL}: TXL-pretreated cardiomyocytes; CM^{TXL}-sEV: sEVs derived from CM^{TXL}; sEV-depleted CM^{TXL}-medium: conditioned medium collected from H/R-treated-CM^{TXL} and depleted of sEVs. All data are mean ± SEM. Statistical analysis was performed with one-way ANOVA followed by Tukey's test.

Figure S11

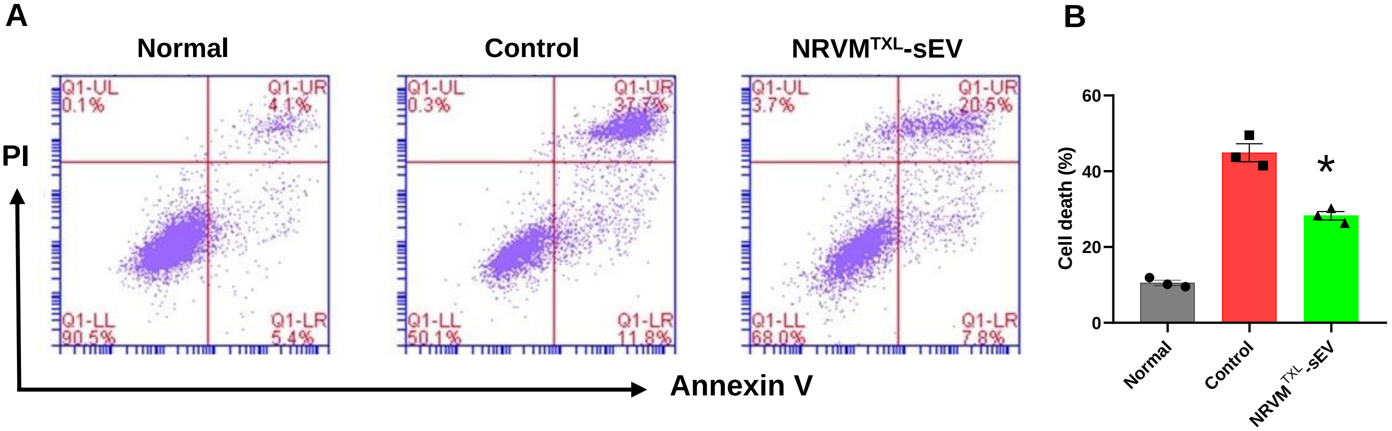

SEVs from TXL-pretreated NRVMs could alleviate H/R-induced injury in RAECs. A) Representative scatter diagram of the death of RAECs with different treatments in flow cytometry assay (n=3). B) Quantitative analysis for dead cells in Flow Cytometry Assay. *P<0.05 vs. Control; sEVs: small extracellular vesicles; NRVMs: neonatal rat ventricular myocytes; RAECs: rat aortic endothelial cells; H/R: Hypoxia 18h/Reoxygenation 2h; $NRVM^{TXL}$: TXL-pretreated NRVMs; $NRVM^{TXL}$-sEV: sEVs derived from $NRVM^{TXL}$. All data are mean ± SEM. Statistical analysis was performed with one-way ANOVA followed by Tukey's test.

Figure S12

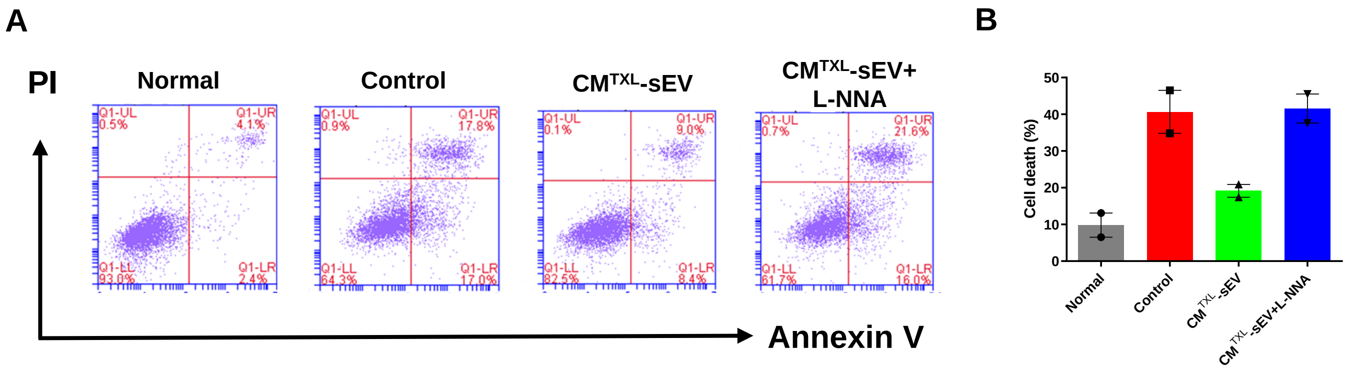

L-NNA, an inhibitor of eNOS, abrogated the protective effects of CM^{TXL}-sEV on CMECs after H/R. A) Representative scatter diagram of the death of CMECs with different treatments in flow cytometry (confirmed in two independent experiments). B) Quantitative analysis for dead cells in Flow Cytometry Assay. sEV: small extracellular vesicles; CMECs: Cardiac microvascular endothelial cells; H/R: Hypoxia/Reoxygenation; CM: cardiomyocyte; CM^{TXL}: TXL-pretreated cardiomyocyte; CM^{TXL}-sEV: sEVs derived from H/R-treated CM^{TXL}

Figure S13

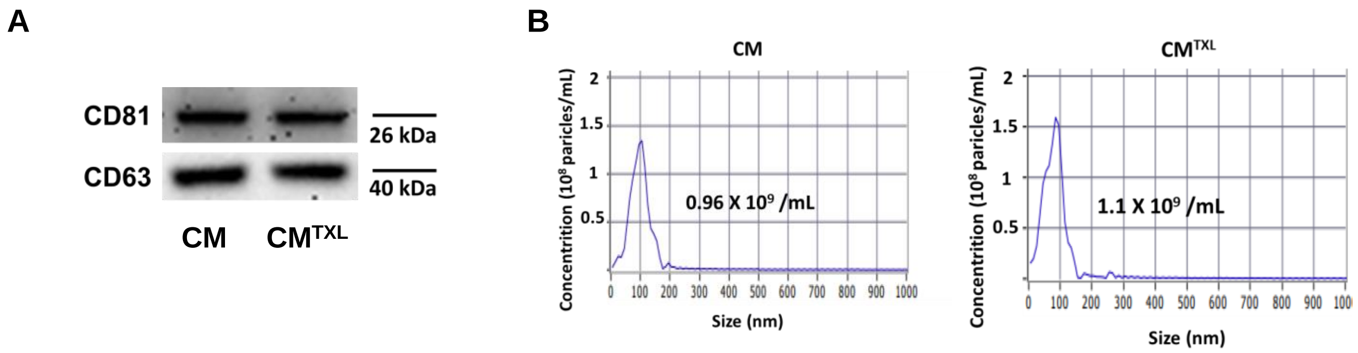

TXL pretreatment had no impacts on sEVs released from CMs. A) Detection of sEV markers with western blotting (n=3); B) Particle number and size distribution measured in nanoparticle tracking analysis were displayed (n=3); sEV: small extracellular vesicle; CM group: sEVs were isolated from conditioned medium of CMs after H/R; CM^{TXL}: sEVs were isolated from conditioned medium of CM^{TXL} after H/R. CM: cardiomyocytes; CM^{TXL}: cardiomyocytes pretreated with TXL for 1 hour

Figure S14

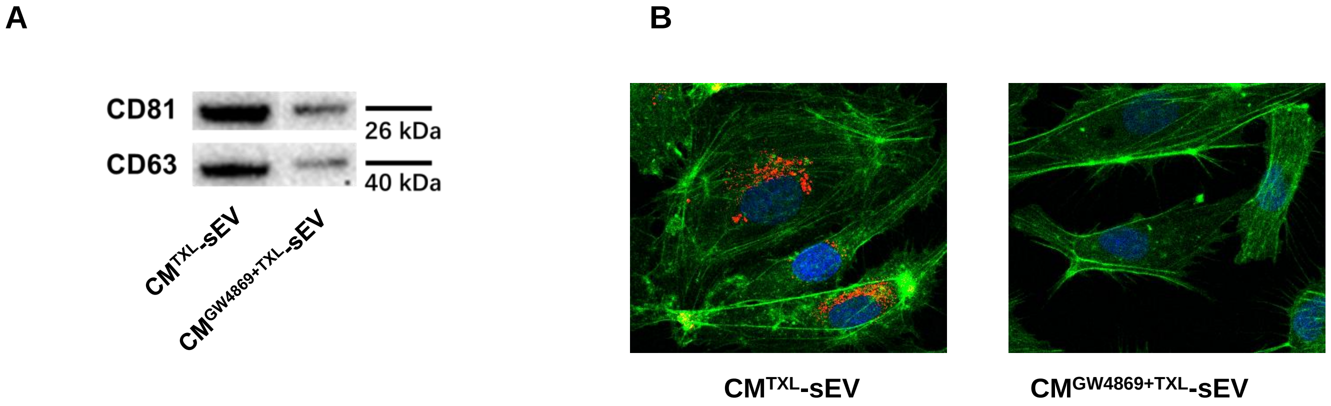

GW4869 could effectively inhibit CM^{TXL} to release sEVs. A) Western blots demonstrating sEV marker in sEVs derived from CMs after H/R (confirmed in two independent experiments). B) Representative confocal images showing sEVs from CMs after H/R were endocytosed by CMECs after 18 hours incubation. Red fluorescence indicates sEVs labelled by dye PKH26. sEVs: small extracellular vesicles; CM^{TXL}-sEV group: sEVs from the conditioned medium of CM^{TXL} after H/R were isolated and then incubated with CMECs for 18 hours; $CM^{GW4869+TXL}$- sEV group: sEVs from the conditioned medium of $CM^{GW4869+TXL}$ after H/R were isolated and then incubated with CMECs for 18 hours. CM: cardiomyocytes; CMECs: Cardiac microvascular endothelial cells; H/R: Hypoxia 18h/Reoxygenation 2h; CM^{TXL}: CM pretreated with TXL for 1 hour; $CM^{GW4869+TXL}$: CM pretreated with GW4869 for 24 hours, and then GW4869 and TXL for 1 hour

Figure S15

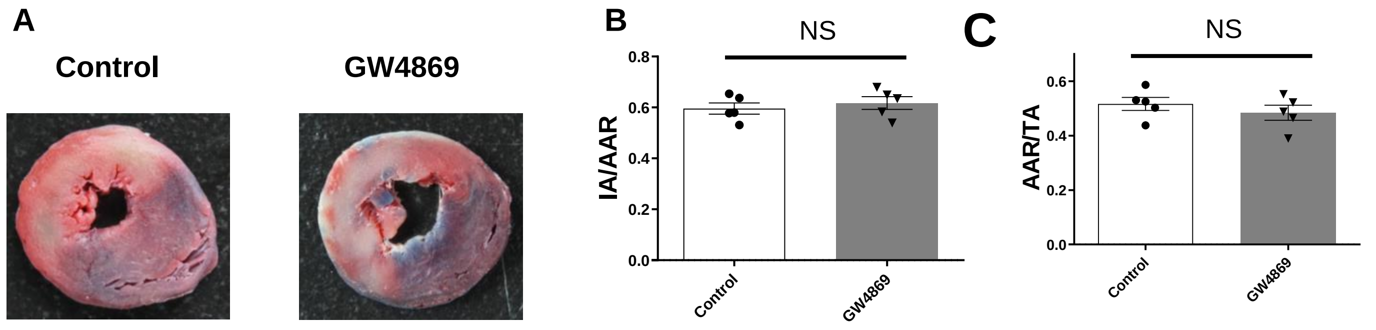

GW4869 alone did not augment infarct size after I/R. A) Representative images for myocardial infraction size assessed by Evans blue/TTC double staining (n=5). B-C) Quantitative analysis for infarct size and area at risk after I/R, respectively. I/R: Ischemia/Reperfusion; IA: infarct area; AAR: area at risk; TA: total area. All data are mean ± SEM. Statistical analysis was performed with unpaired Student T-test.

Figure S16

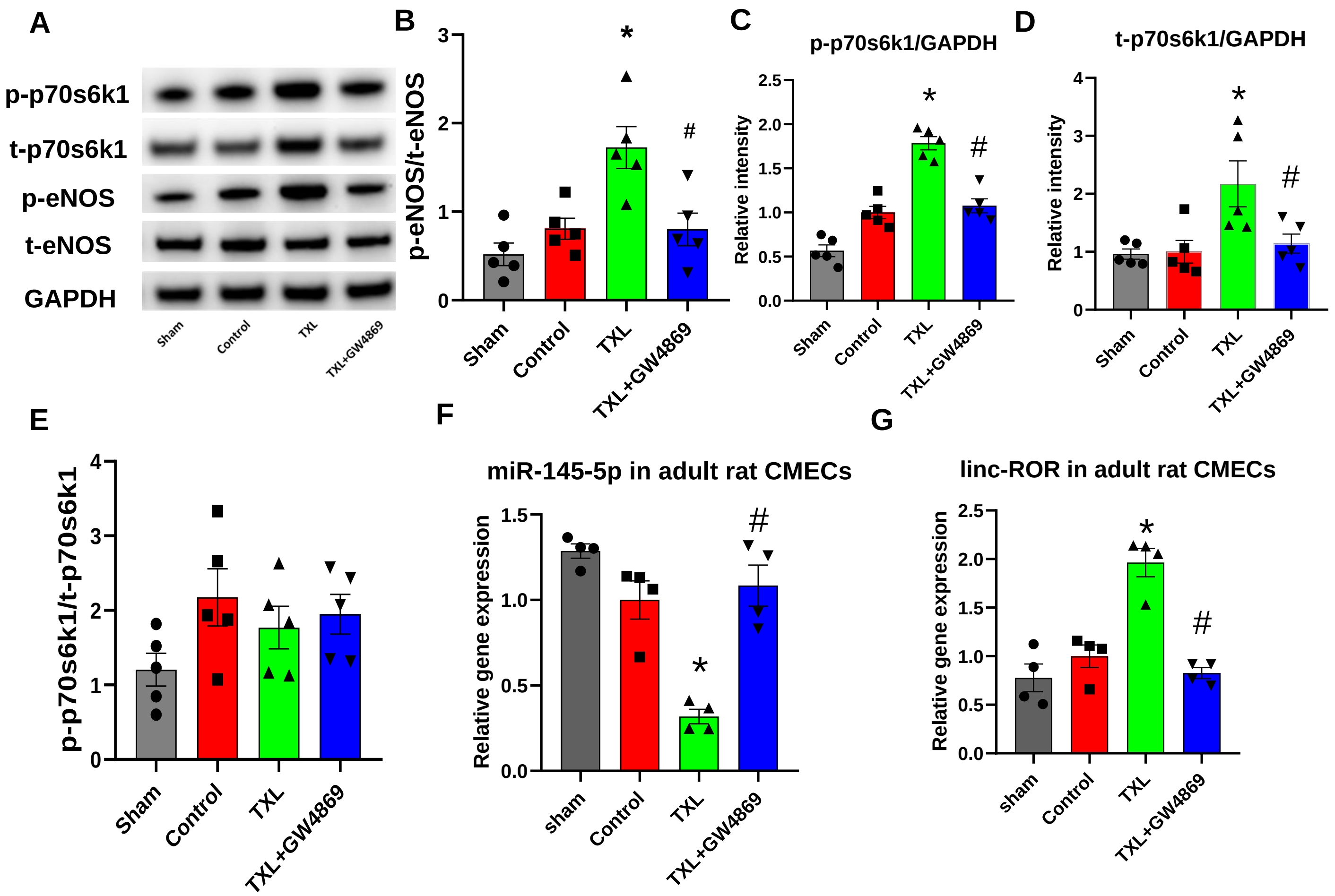

TXL activated the linc-ROR/miR-145-5p/p70s6k1/eNOS pathway in adult rat CMECs during I/R via sEVs. A-E) The levels of p-70s6k1, t-p70s6k1, p-eNOS and t-eNOS were detected by Western Blot (n=5). F) The level of miR-145-5p in CMECs (n=4). G) The level of linc-ROR in CMECs (n=4). * P<0.05 TXL vs. Control; # P <0.05 TXL + GW4869 vs TXL. TXL: Tongxinluo; CMECs: Cardiac microvascular endothelial cells; I/R: Ischemia/Reperfusion; sEVs: small extracellular vesicles; All data are mean ± SEM. Statistical analysis was performed with one-way ANOVA followed by Tukey's test.

Figure S17

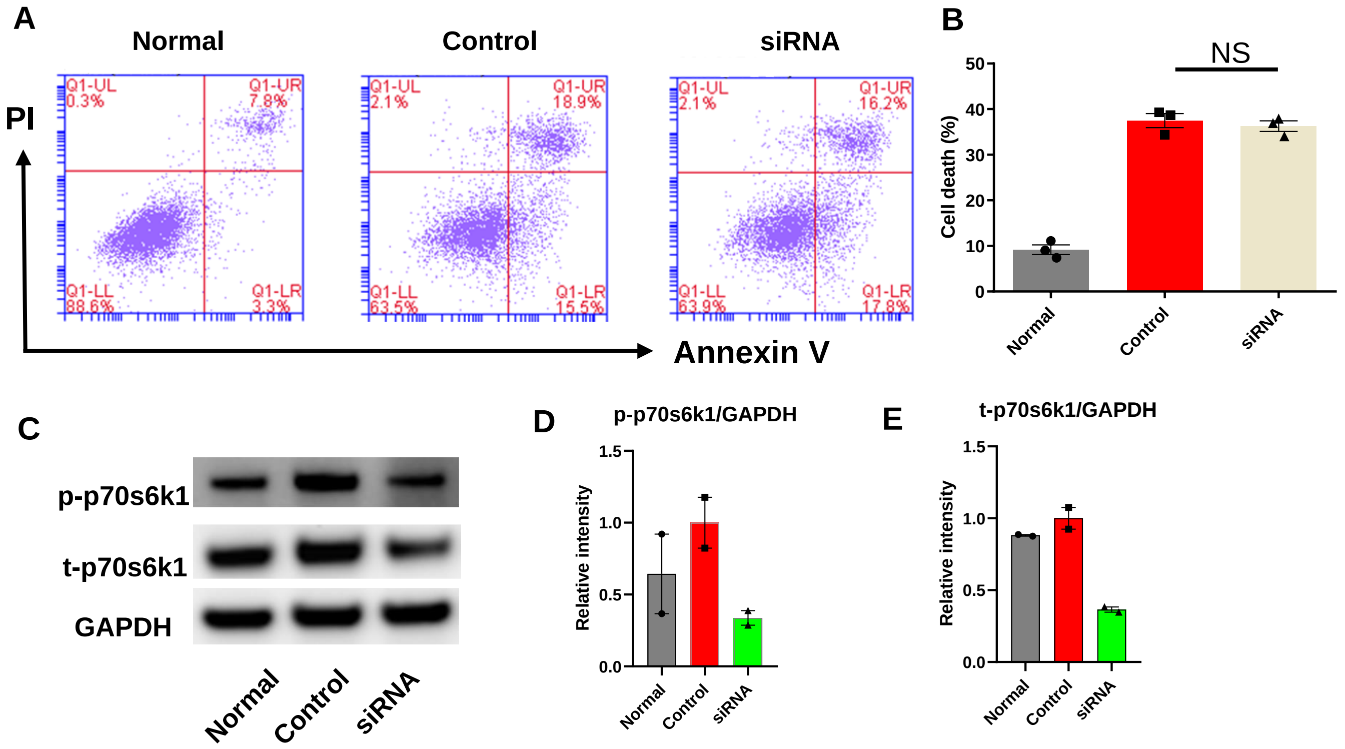

P70s6k1 siRNA alone did not promote the death of CMECs after H/R. A) Representative scatter diagram of the death of CMECs with different treatments in flow cytometry assay (n=3). B) Quantitative analysis for dead cells in Flow Cytometry Assay. C-E) The levels of p-70s6k1 and t-p70s6k1 were detected by Western Blot (2 independent experiments). CMECs: Cardiac microvascular endothelial cells; H/R: Hypoxia/Reoxygenation; siRNA group: CMECs were transfected with p70s6k1 siRNA and then exposed to H/R. All data are mean ± SEM. Statistical analysis was performed with one-way ANOVA followed by Tukey's test.

Figure S18

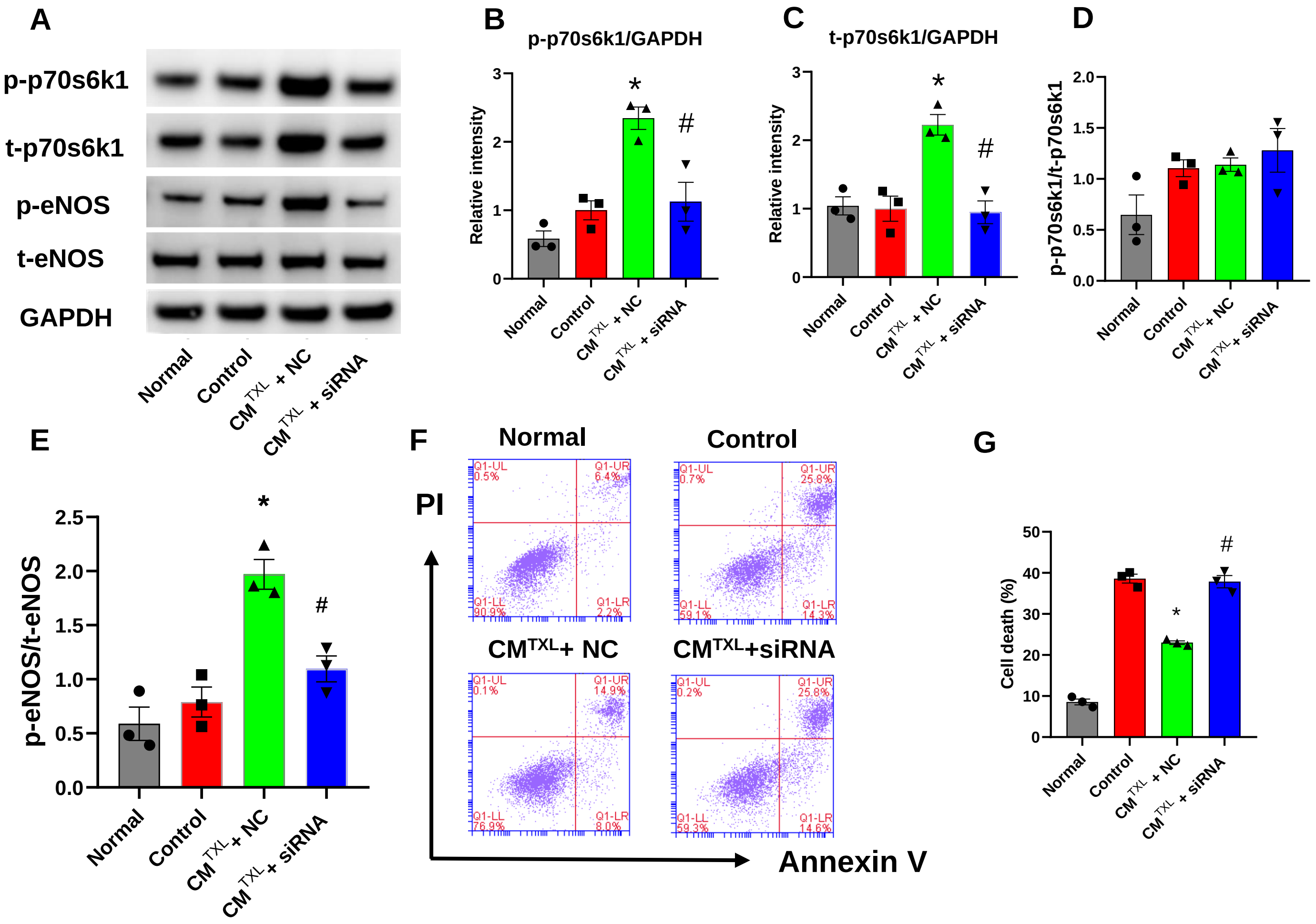

Investigating the role of p70s6k1 in the protective effects of CM^{TXL} sEVs on CMECs with another construct of p70s6k1 siRNA. A-E) The levels of p-70s6k1, t-p70s6k1, p-eNOS and t-eNOS were detected by Western Blot (n=3). F-G) Dead CMECs in flow cytometry assay (n=3). * $P<0.05$ vs. Control; # $P<0.05$ vs CM^{TXL}+NC. sEVs: small extracellular vesicles; CM: cardiomyocyte; CM^{TXL}: TXL-pretreated CMs; CM^{TXL} +siRNA group: CMECs were transfected with p70s6k1 siRNA before being co-cultured with CM^{TXL} and then exposed to H/R; CM^{TXL} +NC group: CMECs were transfected with negative control of siRNA before being co-cultured with CM^{TXL} and then exposed to H/R. All data are mean ± SEM. Statistical analysis was performed with one-way ANOVA followed by Tukey's test.

Figure S19

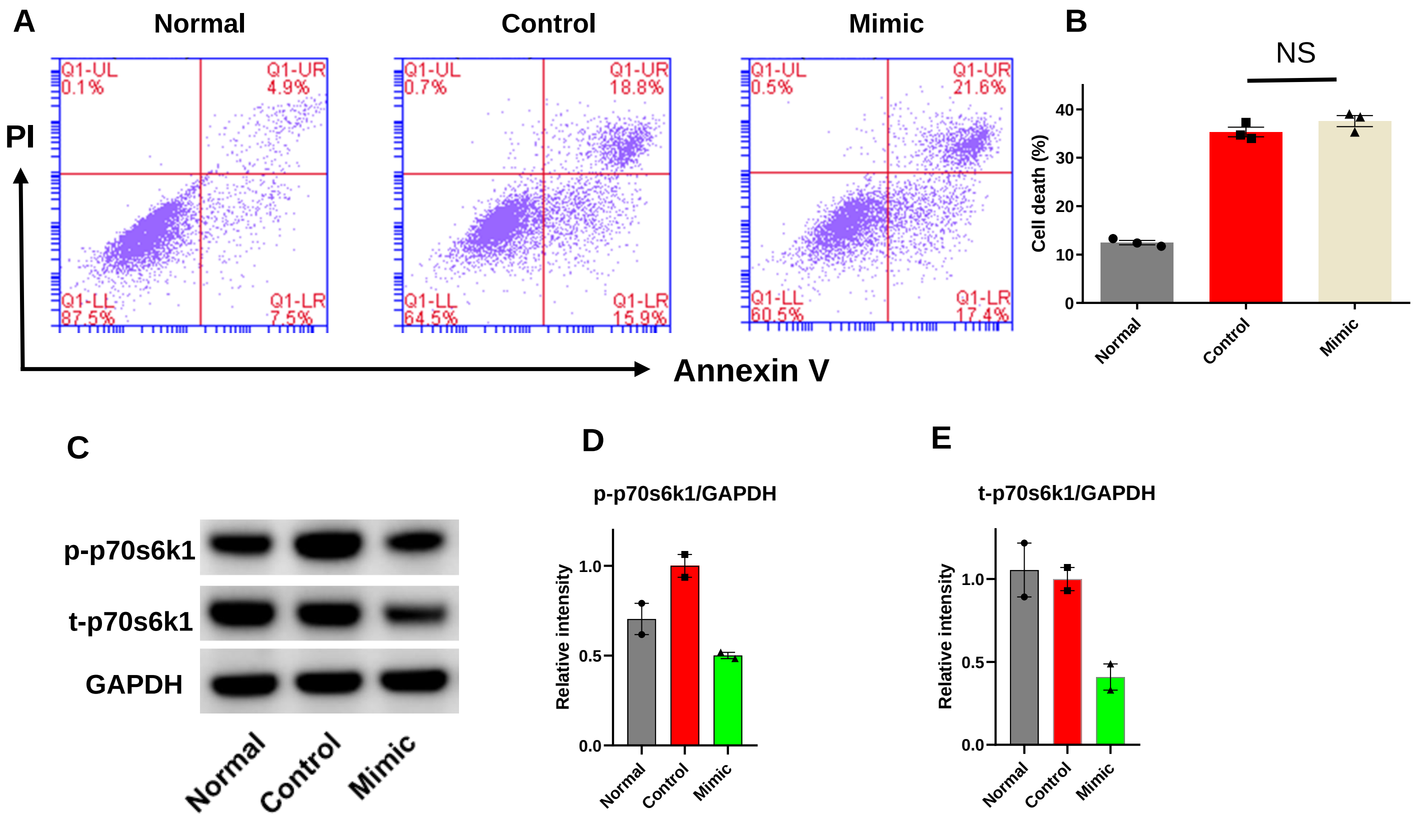

miR-145-5p mimics alone alone did not promote the death of CMECs after H/R. A) Representative scatter diagram of the death of CMECs with different treatments in flow cytometry assay (n=3). B) Quantitative analysis for dead cells in Flow Cytometry Assay. C-E) The levels of p-70s6k1 and t-p70s6k1 were detected by Western Blot (n=2 independent experiments). CMECs: Cardiac microvascular endothelial cells; H/R: Hypoxia/Reoxygenation; Mimic group: CMECs were transfected with miR-145-5p mimics and then exposed to H/R. All data are mean ± SEM. Statistical analysis was performed with one-way ANOVA followed by Tukey’s test.

Figure S20

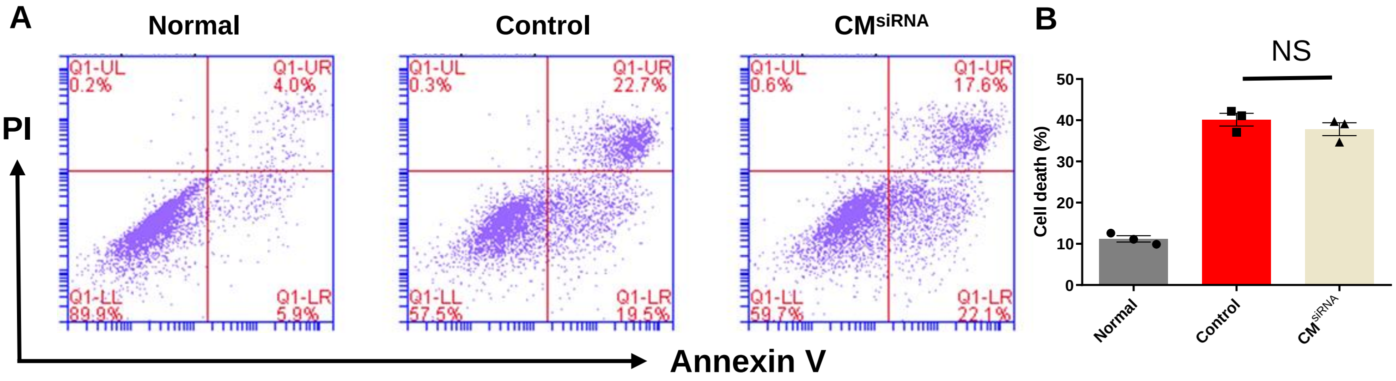

CMs transfected with linc-ROR siRNA did not facilitate the death of CMECs after H/R in the co-culture system. A) Representative scatter diagram of dead CMECs with different treatments in flow cytometry assay (n=3). B) Quantitative analysis for dead cells in Flow Cytometry Assay. CMs: cardiomyocytes; CMECs: Cardiac microvascular endothelial cells; H/R: Hypoxia/Reoxygenation; CM^{siRNA} group: CMECs were co-cultured with CMs having been transfected with linc-ROR siRNA under condition of H/R. All data are mean ± SEM. Statistical analysis was performed with one-way ANOVA followed by Tukey's test.
